# Excess All-Cause Deaths during Coronavirus Disease Pandemic, Japan, January–May 2020^1^

**DOI:** 10.3201/eid2703.203925

**Published:** 2021-03

**Authors:** Takayuki Kawashima, Shuhei Nomura, Yuta Tanoue, Daisuke Yoneoka, Akifumi Eguchi, Chris Fook Sheng Ng, Kentaro Matsuura, Shoi Shi, Koji Makiyama, Shinya Uryu, Yumi Kawamura, Shinichi Takayanagi, Stuart Gilmour, Hiroaki Miyata, Tomimasa Sunagawa, Takuri Takahashi, Yuuki Tsuchihashi, Yusuke Kobayashi, Yuzo Arima, Kazuhiko Kanou, Motoi Suzuki, Masahiro Hashizume

**Affiliations:** Tokyo Institute of Technology, Tokyo, Japan (T. Kawashima);; Keio University, Tokyo (T. Kawashima, S. Nomura, Y. Tanoue, D. Yoneoka, A. Eguchi, S. Shi, Y. Kawamura, H. Miyata);; The University of Tokyo, Tokyo (S. Nomura, D. Yoneoka, S. Shi, M. Hashizume);; Waseda University, Tokyo (Y. Tanoue);; St. Luke’s International University, Tokyo (D. Yoneoka, S. Gilmour);; Chiba University, Chiba, Japan (A. Eguchi);; Nagasaki University, Nagasaki, Japan (C.F.S. Ng); Tokyo University of Science, Tokyo (K. Matsuura);; HOXO-M Inc., Tokyo (K. Matsuura, K. Makiyama, S. Takayanagi);; RIKEN Center for Biosystems Dynamics Research, Osaka, Japan (S. Shi);; National Institute for Environmental Studies, Tokyo (S. Uryu); RIKEN Center for Sustainable Resource Science, Saitama, Japan (Y. Kawamura);; National Institute of Infectious Diseases, Tokyo (T. Sunagawa, T. Takahashi, Y. Tsuchihashi, Y. Kobayashi, Y. Arima, K. Kanou, M. Suzuki)

**Keywords:** coronavirus disease, coronavirus disease, COVID-19, severe acute respiratory syndrome coronavirus 2, SARS-CoV-2, viruses, respiratory infections, zoonoses, excess deaths, all-cause deaths, Japan

## Abstract

To provide insight into the mortality burden of coronavirus disease (COVID-19) in Japan, we estimated the excess all-cause deaths for each week during the pandemic, January–May 2020, by prefecture and age group. We applied quasi-Poisson regression models to vital statistics data. Excess deaths were expressed as the range of differences between the observed and expected number of all-cause deaths and the 95% upper bound of the 1-sided prediction interval. A total of 208–4,322 all-cause excess deaths at the national level indicated a 0.03%–0.72% excess in the observed number of deaths. Prefecture and age structure consistency between the reported COVID-19 deaths and our estimates was weak, suggesting the need to use cause-specific analyses to distinguish between direct and indirect consequences of COVID-19.

Severe acute respiratory syndrome coronavirus 2 (SARS-CoV-2) first appeared in December 2019 in Wuhan, China, and has rapidly led to a global pandemic (*1*). Globally, accurate figures on deaths caused by coronavirus disease (COVID-19) have been difficult to obtain because of limited availability and quality of virus testing (*2*,*3*) (Y. Yang et al., unpub. data, https://www.medrxiv.org/content/10.1101/2020.02.11.20021493v2); it is generally accepted that many deaths caused by COVID-19 have not yet been recorded (*4*). Lockdown measures are in place in many countries and regions around the world, but such measures can lead to reduced access to health services, exacerbating chronic diseases and delaying response to acute diseases (*5*). Access to hospitals for elective surgery may also be hampered by the collapsing medical system associated with the increased number of COVID-19 patients (*6*). The cause of death, especially among elderly persons in care homes or living alone, may not be adequately diagnosed or even recorded during a pandemic situation (*7*).

When comprehensive testing is lacking, the mortality burden of a new pandemic is commonly estimated by an increase in the number of deaths that is greater than would be expected under normal circumstances (e.g., in the absence of a pandemic)—the so-called excess-death approach (*8*,*9*). This approach can be applied to specific causes of death directly related to the pathogen, such as for pneumonia or other respiratory diseases, or to other categories of death that are directly or indirectly affected by a pandemic. For example, excess-death methods have been used to quantify formal underestimation of the mortality burden of COVID-19 in many heavily affected countries (*10*–*17*).

The early and comprehensive response to the COVID-19 pandemic in Japan probably enabled the country to avoid the severe epidemics experienced in Europe; as of September 2, 2020, a total of 68,392 COVID-19 cases and 1,296 related deaths had occurred in Japan (*18*). Nonetheless, despite an effective response, in early April as cases began to increase rapidly, the health system began to experience pressures similar to those in other countries, and the government declared a state of emergency (*19*). Perhaps uniquely among global movement restrictions, in Japan, these restrictions were completely voluntary, with no legal force; routine healthcare functions continued, including elective surgery and outpatient services for nonurgent health issues. Given the relatively limited spread of the epidemic in Japan and the voluntary nature of the lockdown, it is possible that the pattern of excess deaths in Japan differs from that in other countries. To provide insight into the mortality burden of COVID-19 in Japan, we estimated excess deaths from all causes during each week from the early COVID‐19 outbreak in Japan, January–May 2020, by prefecture and patient age. Ethics approval was granted by the ethics committee of the National Institute of Infectious Diseases (authorization no. 1174).

## Methods

### Data

For this study, we used mortality data from the Vital Statistics System of Japan, which compiles the Vital Statistics Survey data prepared by each municipality under the Family Registration Law and the Provisions on the Notification of Stillbirths (*20*). Vital statistics are divided into 3 major types: annual vital statistics, monthly vital statistics, and prompt vital statistics. Annual vital statistics are compiled for 1 year (January–December) from the monthly vital statistics and are published around September each year. Monthly vital statistics are published ≈5 months after the month in which the survey forms are collected from the municipalities. Prompt vital statistics are published ≈2 months after the month of survey form collection.

According to the Family Registration Law, a notification of death must be submitted to a municipal office within 7 days of the day on which the person’s death was confirmed. The notification must be submitted by a relative or a person who lived with the deceased or, in some cases, by landlords, house managers, or persons with similar roles. For the Prompt Vital Statistics report, data for a given month are based on death notifications reported to the municipality by the 14th of the following month. In other words, a death notification reported on or after the 15th with a death date of the previous month is placed in the dataset for the current month, not the previous month. For example, if a death notification is reported by February 14 with a death date of January 20, the data will be included in the January Prompt Vital Statistics report, but if the death is reported on February 15, it will be included in the February Prompt Vital Statistics report, referred to as a reporting delay. The delay in reporting deaths addressed in this study refers to any delays between the death confirmation process to submission of the death notifications to the municipal offices, perhaps depending on where the death occurs. The observed numbers of deaths in the Prompt Vital Statistics report were adjusted for this reporting delay up to 3 months to avoid a possible undercount of observed deaths. We used these adjusted data in our excess deaths analysis. 

For this analysis, we used data from 2012 on (including the last few days of 2011 for weekly analysis purposes): Annual Vital Statistics report for 2011–2018, Monthly Vital Statistics report for 2019, and Prompt Vital Statistics report for January–May 2020. The target population was all persons who had resident cards and died in Japan, regardless of nationality. However, the analysis excluded those who died abroad, those who were staying in Japan for a short time (without a resident card), and those whose place of residence or date of birth was unknown. Our data did not include cause-of-death information; only age at death and place of residence (prefecture) were available for analysis.

### Excess Deaths Analysis

To estimate excess deaths in Japan, we used the Farrington algorithm, which is commonly used to estimate excess deaths and is used by the US Centers for Disease Control and Prevention to estimate excess deaths associated with COVID-19 (*21*). The Farrington algorithm uses a quasi-Poisson regression (a generalized linear model accounting for overdispersion) to estimate the expected number of deaths per week. The algorithm is designed to limit the data used for estimation: the expected number of deaths at a certain week *t* is estimated by using only the data during *t* − *w* and *t* + *w* weeks of years *h* − *b* and *h* − 1, where *w* and *b* are predetermined parameters and *h* is the year of *t*, referred to as the reference period. Data for a period of 1 year that is not included in the reference period are divided equally and included in the regression model as dummy variables, which enables the model to capture seasonality. Thus, the regression model is log(*E*(*Y_t_*) = α + β*t* + *f^T^*(*t*)γ*_f_*_(_*_t_*_)_, where *Y_t_* is the number of deaths at a certain week *t*, α and β are regression parameters, γ*_f_*_(_*_t_*_)_is a regression parameter vector representing seasonality, and *f*(*t*) is a vector of dummy variables that equally divides the time points outside the reference period. 

In this study, we divided the data into 9 periods, as was done in a previous study (*21*). More details can be found elsewhere (*8*,*9*). In our study, we considered data up to 5 years ago (*b* = 5) and used data for 3 weeks (*w* = 3) before and after a certain point as the reference period, as was done in previous studies (*21*,*22*). We checked for overdispersion by comparing mean and variance of weekly deaths and used an overdispersed Poisson model where significant overdispersion was found after a regression-based (1-sided) test for overdispersion in the Poisson model (*23*). Also, as a sensitivity analysis, we changed the reference period to confirm the robustness of the results based on combinations of *b* = 3 or 4 and *w* = 2 or 4.

The model estimation was stratified by prefecture and age group (all ages, <25 years, 25–44 years, 45–64 years, 65–74 years, 75–84 years, >85 years). Age group was determined by considering the age structure and the number of persons sufficient for analysis. All age estimates (for all persons) do not add up to age group–specific estimates. The conversion from daily data to weekly data is based on the epidemiologic week of the National Institute of Infectious Diseases’ Infectious Diseases Weekly Report (*24*).

### Number of Excess All-Cause Deaths

On the basis of the model equation shown in the previous section, we estimated the expected number of all-cause deaths per week and the associated 95% upper bound of the 1-sided prediction interval, which is an indicator of uncertainty. We set these 2 thresholds (point estimate and upper bound) for excess death according to previous studies (*21*). We report the range of differences between the observed number of all-cause deaths and each of these thresholds as excess deaths.

To obtain the national level of excess all-cause deaths for each week, we summed the observed and the expected number of all-cause deaths separately across all prefectures in each week and computed the weekly differences for the country. The total (cumulative) number of excess all-cause deaths in each prefecture during the COVID-19 pandemic was calculated by summing the excess all-cause deaths (with negative values set to 0) in each week, from the beginning of 2020 (December 30, 2019–January 5, 2020) through May 2020 (May 25–31, 2020). We calculated the national cumulative number of excess all-cause deaths for the given period by summing the prefecture-specific excess deaths, a method consistent with US Centers for Disease Control and Prevention methods used (*8*). Last, we defined the percentage of excess deaths during the COVID-19 pandemic as the cumulative number of excess deaths divided by the observed cumulative number of deaths.

### Adjusting for Reporting Delays

The observed number of deaths in the Prompt Vital Statistics report may differ from the actual number of deaths because of delays in reporting deaths (i.e., fewer deaths in the Prompt Vital Statistics report than in Monthly or Annual Vital Statistics reports published later). We took into account the reporting delay of up to 3 months by calculating the reporting delay rate (deaths reported 1, 2, and 3 months behind) for each prefecture and then adjusting the observed number of deaths in the latest 3 months (March–May 2020). Thus, the observed number of deaths in March was adjusted for a 3-month reporting delay (such as June deaths not available in our data; similarly, those in April were adjusted for 2- and 3-month reporting delays and those in May were adjusted for 1-, 2-, and 3-month reporting delays (Appendix).

To verify the validity of this adjustment method, we compared the weekly observed number of all-cause deaths in February, based on the Prompt Vital Statistics report through May with no adjustment for the reporting delay, and those in February, based on the Prompt Vital Statistics report through April with adjustment for reporting delays in May. The proportionate differences were then calculated for each week of February 2020. The largest difference was observed in Tokyo Prefecture (January 27–February 2, 2020) and Fukuoka Prefecture (January 27–February 2, 2020) at 5 deaths (Appendix Table 1), and the largest proportionate difference was observed in Tottori Prefecture (January 27–February 2, 2020) at 0.694%.

## Results

We calculated mean and variance of the outcome (i.e., no. deaths/week among age- and prefecture-combined populations) to test the overdispersion; on the basis of the results (p<0.01), we used the quasi-Poisson regression for analysis. The cumulative number of excess all-cause deaths of the 47 prefectures was 208–4,322 (0.03%–0.72% excess) (Table). Weeks with observed all-cause deaths exceeding the 95% upper bound of the 1-sided interval of predicted deaths from the beginning of 2020 through May 2020 were detected in 13 prefectures. The cumulative numbers of excess all-cause deaths (percent excess) over the period for the 13 prefecture were as follows: Ibaraki, 1–87, 0.01%–0.60%; Tochigi, 13–137, 0.14%–1.42%; Gunma, 31–146, 0.30%–1.43%; Saitama, 14–334, 0.05%–1.10%; Chiba, 51–253, 0.19%–0.94%; Tokyo, 32–330, 0.06%–0.63%; Toyama, 18–120, 0.32%–2.11%; Shizuoka, 2–109, 0.01%–0.59%; Aichi, 7–214, 0.02%–0.70%; Osaka, 6–277, 0.01%–0.69%; Nara, 21–107, 0.32%–1.65%; Tokushima, 4–71, 0.09%–1.64%; and Kagawa, 8–135, 0.15%–2.51%.

**Table:** Number of observed and excess all-cause deaths, and reported number of COVID-19 deaths, Japan, December 30, 2019–May 31, 2020*

Prefecture	No. all-cause deaths			No. COVID-19 deaths	No. tests
	Observed	Excess	Percentage		
Hokkaido	27,661	0–115	0.00–0.42	86	14,000
Aomori	7,713	0–36	0.00–0.47	1	850
Iwate	7,588	0–81	0.00–1.07	0	662
Miyagi	10,725	0–57	0.00–0.53	1	2,944
Akita	6,656	0–72	0.00–1.08	0	933
Yamagata	6,606	0–49	0.00–0.74	0	2,659
Fukushima	10,714	0–34	0.00–0.32	0	4,452
Ibaraki	14,443	1–87	0.01–0.60	10	4,628
Tochigi	9,623	13–137	0.14–1.42	0	3,871
Gunma	10,175	31–146	0.30–1.43	19	3,655
Saitama	30,426	14–334	0.05–1.10	48	20,735
Chiba	26,841	51–253	0.19–0.94	45	14,688
Tokyo	52,350	32–330	0.06–0.63	305	38,566
Kanagawa	36,174	0–89	0.00–0.25	82	9,446
Niigata	12,704	0–0	0.00–0.00	0	4,180
Toyama	5,689	18–120	0.32–2.11	22	3,144
Ishikawa	5,538	0–33	0.00–0.60	25	2,723
Fukui	4,045	0–47	0.00–1.16	8	2,631
Yamanashi	4,276	0–60	0.00–1.40	1	3,877
Nagano	11,148	0–29	0.00–0.26	0	2,714
Gifu	9,889	0–31	0.00–0.31	7	3,610
Shizuoka	18,554	2–109	0.01–0.59	1	3,521
Aichi	30,583	7–214	0.02–0.70	34	9,970
Mie	9,056	0–57	0.00–0.63	1	2,505
Shiga	5,606	0–65	0.00–1.16	1	1,856
Kyoto	11,814	0–84	0.00–0.71	17	7,933
Osaka	40,017	6–277	0.01–0.69	83	31,156
Hyogo	25,490	0–69	0.00–0.27	42	11,128
Nara	6,474	21–107	0.32–1.65	2	2,545
Wakayama	5,547	0–66	0.00–1.19	3	3,701
Tottori	3,156	0–44	0.00–1.39	0	1,338
Shimane	4,203	0–73	0.00–1.74	0	1,125
Okayama	9,493	0–75	0.00–0.79	0	1,705
Hiroshima	13,250	0–45	0.00–0.34	3	6,907
Yamaguchi	8,171	0–50	0.00–0.61	0	1,701
Tokushima	4,339	4–71	0.09–1.64	1	741
Kagawa	5,374	8–135	0.15–2.51	0	2,187
Ehime	7,913	0–50	0.00–0.63	4	2,074
Kochi	4,383	0–58	0.00–1.32	3	1,793
Fukuoka	23,346	0–77	0.00–0.33	26	12,634
Saga	4,412	0–53	0.00–1.20	0	1,417
Nagasaki	7,686	0–85	0.00–1.11	1	2,754
Kumamoto	9,340	0–43	0.00–0.46	3	3,924
Oita	6,279	0–52	0.00–0.83	1	3,988
Miyazaki	6,101	0–120	0.00–1.97	0	1,368
Kagoshima	9,309	0–59	0.00–0.63	0	1,859
Okinawa	5,334	0–44	0.00–0.82	6	2,863
Total	596,214	208–4,322	0.03–0.72	892	269,661

*The national-level cumulative number of excess all-cause deaths was calculated by summing the excess all-cause deaths of 47 prefectures (*25*). COVID-19, coronavirus disease.

Of the 32 prefectures in which COVID-19 deaths were confirmed through end of May 2020, the observed number of all-cause deaths for all ages exceeded the 95% upper bound in 11 prefectures (34.4%, 11 of 32) for some weeks and the point estimates in all prefectures. Of the remaining 15 prefectures in which no deaths from COVID-19 had been confirmed, the observed number of all-cause deaths for all ages exceeded the 95% upper bound in 2 (13.3%) of the 15 prefectures for some weeks and the point estimate in 14 (93.3%) of the 15 prefectures. Only in Niigata Prefecture did the observed number of all-cause deaths for all ages not exceed the point estimates for the period.

Sensitivity analyses in which the reference period was changed to confirm the robustness of the results showed that, depending on the model parameter settings, weeks with observed all-cause deaths exceeding the 95% upper bound were also observed in additional prefectures, including Shiga, Shimane, Kochi, Fukuoka, Kumamoto, Oita, and Miyazaki (Appendix Table 2). As of the end of May 2020, deaths from COVID-19 had not been confirmed in Shimane and Miyazaki Prefectures.

The totals of excess all-cause deaths (percent excess) at the national level by age group were as follows: <25 years of age, 47–751 (1.61–25.76); 25–44 years, 66–1,302 (0.84–16.66); 45–64 years, 207–2,958 (0.47–6.67); 65–74 years, 143–2,959 (0.17–3.48); 75–84 years, 110–3,100 (0.07–1.86); and >85 years, 73–2,466 (0.03–0.85) (Appendix Table 3). Weeks with observed all-cause deaths exceeding the 95% upper bound for each age group were observed in 28, 23, 25, 25, 20, and 8 prefectures for these age groups, respectively. Weeks in which observed all-cause deaths exceeded point estimates were observed for all 47 prefectures and age groups.

Weekly observed and expected number of all-cause deaths in 4 prefectures reflect the large number of reported COVID19 deaths as of the end of May 2020 (Tokyo, Hokkaido, Osaka, and Kanagawa) for all ages and by age group (Appendix Figure 1). For all age groups, weeks of excess deaths occurred in previous years, not only during the COVID-19 pandemic. Data for the other 43 prefectures and national-level data are also shown (Appendix Figure 2).

## Discussion

Excess-death monitoring has been used to track influenza epidemics worldwide and to identify the high potential mortality burden of COVID-19 in some hard-hit countries. We used a similar approach to capture the overall mortality burden of COVID-19. Monitoring changes and trends in all-cause deaths provides insight into the magnitude of the overall mortality burden caused by COVID-19, both directly and indirectly, which was overlooked in the official number of COVID-19 deaths. Given the variability in testing intensity among prefectures, this type of monitoring provides valuable information about the social effects of a pandemic and the extent to which virus testing may miss deaths caused by COVID-19. Useful indicators of the severity of the pandemic may include syndromic endpoints such as COVID-19 deaths, outpatient visits, and emergency department visits for fever or other COVID-19–associated symptoms (*26*). However, in the absence of comprehensive testing for COVID-19, estimates of the number of excess all-cause deaths may be more reliable than the reported number of COVID-19 deaths, especially in areas where testing is not widespread, so as to assess the progression of a pandemic and the effects of interventions.

During January–May 2020, the 208–4,322 excess deaths in all 47 prefectures represented just 0.03%–0.72% of all deaths observed in Japan through May 31, 2020. Although a complete country comparison is not possible, given the different methods for estimating excess deaths in each country (*2*,*3*) (Y. Yang et al., unpub. data, https://www.medrxiv.org/content/10.1101/2020.02.11.20021493v2), the number of deaths caused by COVID-19 in Japan, which was ≈0.7 deaths/100,000 population as of May 31 (and 1.3 deaths/100,000 population as of October 31), is 10 to 100 times lower than that for many countries in Europe and for the United States (*27*), indicating the relative low overall mortality burden from COVID-19 in Japan. This low overall mortality burden probably reflects the benefits of Japan’s rapid and comprehensive response to the COVID-19 pandemic, which began with voluntary restrictions of public events in mid-February 2020 (*28*).

The excess all-cause deaths that we report can be interpreted as the sum of the following scenarios: 1) COVID-19 was the primary cause of death; 2) although other causes were diagnosed as the primary cause of death, the actual cause of death was COVID-19; 3) COVID-19 was not diagnosed as the primary cause of death, but because of the effects of the COVID-19 epidemic, death was caused by other diseases. For example, persons may hesitate to visit a hospital because of the declaration of an emergency or self-restraint in going out, or their chronic disease may worsen because of lifestyle changes, resulting in death (*21*). On the other hand, if deaths from causes other than COVID-19 decrease under the pandemic situation (as may have occurred with deaths from traffic accidents and suicide [*29*]), excess deaths directly caused by COVID-19 may be offset by the negative portion of those deaths. In fact, traffic accidents in Japan had decreased because of decreased traffic volume resulting from stay-at-home requests by central and local governments, and it is possible that the number of deaths from injuries had decreased (*29*).

Weeks with observed all-cause deaths exceeding the 95% upper bound of the 1-sided prediction interval were observed for 13 prefectures, of which COVID-19 deaths have been confirmed for 11. On the other hand, COVID-19 deaths have been observed in 22 other prefectures where no all-cause excess deaths were observed, suggesting that COVID-19 deaths in these prefectures were not high enough to overcome natural weekly variations in mortality rates, that there may be an offsetting reduction in deaths because of the indirect effects of the pandemic in these communities, or both. 

According to Ministry of Health, Labour and Welfare data as of May 27, 2020, proportions of COVID-19 deaths were higher for persons in older age groups: 55.8% at >80 years of age, followed by 27.3% at 70–79 years (*30*). Although the officially reported number of COVID-19 deaths may not be free of bias (e.g., different likelihood of testing by age group), these data indicate that prefecture and age structure of the reported COVID-19 deaths were not consistent with our estimates, suggesting the need to distinguish between direct and indirect consequences of COVID-19 by using cause-specific analyses. For the design of future broad-based infectious disease countermeasures such as lockdowns, knowing whether excess deaths in vulnerable age groups arises from direct COVID-19 deaths, indirect causes, or preventable deaths from unrelated causes would be useful.

The limitations of our analysis are the same as those for other excess-deaths studies (*15*,*31*). First, for this study, we did not take into account the cause of death, so the excess death estimates we present are not necessarily estimates of excess deaths caused by COVID-19. In addition, data from January–May 2020 are incomplete in the Prompt Vital Statistics report, especially in the most recent month. We have not considered the cause of the delay in reporting (e.g., delay mechanism) because we believe that adjusting for the delay by cause was impossible. Therefore, we selected a comprehensive adjustment method that does not depend on the cause of the delay by setting 3 assumptions (Appendix). We have also confirmed that validity is sufficient. Validity evaluation indicated that our adjustment for the reporting delay was reasonable to some extent, although this evaluation is within the scope of our 3 assumptions. Although waiting until Monthly Vital Statistics reports are published before analyzing the complete data would be ideal, during a public health emergency it is necessary to analyze the data in a timely manner and the limitations of data adjustment are a trade-off. Last, the method we used in this study is an algorithm for identifying excess deaths, which was not designed for assessing death reduction (*8*,*9*). If the expected number of deaths in a week was less than the actual number of deaths (negative value), the negative value was set to 0. However, as noted above, the effect of COVID-19 on mortality burden has not necessarily been positive (an increasing effect) but may be negative (a decreasing effect). For example, no deaths caused by COVID-19 were observed in Niigata Prefecture as of May 2020, and this study estimated 0–0 excess deaths in the prefecture during January–May 2020. In prefectures where the effect of COVID-19 is relatively small, an algorithm that identifies exiguous deaths might provide more suggestive data than an algorithm that identifies excess deaths. However, our aim with this study was to evaluate the increase in the mortality burden caused by COVID-19, using the methods of previous studies conducted in other countries; exiguous deaths will be evaluated in future analyses.

In conclusion, we found a much lower overall excess mortality burden from COVID-19 in Japan than in Europe and the United States. However, a weak prefecture and age structure consistency between the reported COVID-19 deaths and our estimates also suggest the need to distinguish between direct and indirect consequences of COVID-19 by cause-specific analyses, which can provide more information about the severity and progression of the COVID-19 pandemic. More detailed cause-specific analyses of excess deaths in Japan, especially among persons in older age groups, will enable better design of future interventions to protect vulnerable age groups and also offer lessons to other countries on proper management and implementation of movement restrictions. By paying careful attention to the excess death patterns in Japan, countries more heavily affected by COVID-19 can improve their own future response and better respond to the health needs of critically affected countries.

Appendix Additional results from study of excess all-cause deaths during coronavirus disease pandemic, Japan, January–May 2020.
