# Supplementary material for: Excess All-Cause Deaths during Coronavirus Disease Pandemic, Japan, January–May 2020
Source: Emerg Infect Dis. 2021 Mar;27(3):789–95. doi: 10.3201/eid2703.203925 (PMC7920666; doi:10.3201/eid2703.203925)
Supplement: Appendix — Additional results from study of excess all-cause deaths during coronavirus disease pandemic, Japan, January–May 2020. [file 20-3925-Techapp-s1.pdf]

Article DOI: https://doi.org/10.3201/eid2703.203925

Excess All-Cause Deaths during Coronavirus Disease Pandemic, Japan, January–May 2020

Appendix

Appendix Table 1. Validity test results for reporting delays adjustment.

Area	Week ending date	Difference*	Percent difference, %
Hokkaido	February 2, 2020	1	0.074
Hokkaido	February 9, 2020	-1	-0.078
Hokkaido	February 16, 2020	2	0.148
Hokkaido	February 23, 2020	2	0.165
Aomori	February 2, 2020	0	0.000
Aomori	February 9, 2020	0	0.000
Aomori	February 16, 2020	1	0.292
Aomori	February 23, 2020	0	0.000
Iwate	February 2, 2020	0	0.000
Iwate	February 9, 2020	-1	-0.264
Iwate	February 16, 2020	0	0.000
Iwate	February 23, 2020	-1	-0.271
Miyagi	February 2, 2020	0	0.000
Miyagi	February 9, 2020	0	0.000
Miyagi	February 16, 2020	1	0.191
Miyagi	February 23, 2020	0	0.000
Akita	February 2, 2020	0	0.000
Akita	February 9, 2020	0	0.000
Akita	February 16, 2020	0	0.000
Akita	February 23, 2020	0	0.000
Yamagata	February 2, 2020	0	0.000
Yamagata	February 9, 2020	0	0.000
Yamagata	February 16, 2020	0	0.000
Yamagata	February 23, 2020	0	0.000
Fukushima	February 2, 2020	-1	-0.199
Fukushima	February 9, 2020	-1	-0.187
Fukushima	February 16, 2020	-1	-0.190
Fukushima	February 23, 2020	-1	-0.182
Ibaraki	February 2, 2020	1	0.137
Ibaraki	February 9, 2020	-1	-0.139
Ibaraki	February 16, 2020	-1	-0.138
Ibaraki	February 23, 2020	-1	-0.155
Tochigi	February 2, 2020	0	0.000
Tochigi	February 9, 2020	0	0.000
Tochigi	February 16, 2020	1	0.209
Tochigi	February 23, 2020	1	0.234
Gunma	February 2, 2020	-1	-0.222
Gunma	February 9, 2020	-1	-0.202
Gunma	February 16, 2020	-1	-0.207
Gunma	February 23, 2020	-1	-0.210
Saitama	February 2, 2020	3	0.198
Saitama	February 9, 2020	-3	-0.219
Saitama	February 16, 2020	1	0.068
Saitama	February 23, 2020	-2	-0.148
Chiba	February 2, 2020	0	0.000
Chiba	February 9, 2020	-1	-0.082

Area	Week ending date	Difference*	Percent difference, %
Chiba	February 16, 2020	0	0.000
Chiba	February 23, 2020	-1	-0.084
Tokyo	February 2, 2020	5	0.195
Tokyo	February 9, 2020	-2	-0.083
Tokyo	February 16, 2020	4	0.161
Tokyo	February 23, 2020	2	0.083
Kanagawa	February 2, 2020	3	0.167
Kanagawa	February 9, 2020	-3	-0.170
Kanagawa	February 16, 2020	1	0.054
Kanagawa	February 23, 2020	0	0.000
Niigata	February 2, 2020	0	0.000
Niigata	February 9, 2020	0	0.000
Niigata	February 16, 2020	1	0.168
Niigata	February 23, 2020	0	0.000
Toyama	February 2, 2020	-1	-0.415
Toyama	February 9, 2020	-1	-0.395
Toyama	February 16, 2020	0	0.000
Toyama	February 23, 2020	-1	-0.342
Ishikawa	February 2, 2020	0	0.000
Ishikawa	February 9, 2020	0	0.000
Ishikawa	February 16, 2020	0	0.000
Ishikawa	February 23, 2020	0	0.000
Fukui	February 2, 2020	0	0.000
Fukui	February 9, 2020	0	0.000
Fukui	February 16, 2020	0	0.000
Fukui	February 23, 2020	0	0.000
Yamanashi	February 2, 2020	0	0.000
Yamanashi	February 9, 2020	0	0.000
Yamanashi	February 16, 2020	1	0.478
Yamanashi	February 23, 2020	0	0.000
Nagano	February 2, 2020	-1	-0.189
Nagano	February 9, 2020	-1	-0.187
Nagano	February 16, 2020	-1	-0.182
Nagano	February 23, 2020	-1	-0.180
Gifu	February 2, 2020	0	0.000
Gifu	February 9, 2020	-1	-0.217
Gifu	February 16, 2020	-1	-0.200
Gifu	February 23, 2020	0	0.000
Shizuoka	February 2, 2020	-1	-0.111
Shizuoka	February 9, 2020	-1	-0.112
Shizuoka	February 16, 2020	-1	-0.107
Shizuoka	February 23, 2020	0	0.000
Aichi	February 2, 2020	1	0.067
Aichi	February 9, 2020	-1	-0.067
Aichi	February 16, 2020	2	0.134
Aichi	February 23, 2020	0	0.000
Mie	February 2, 2020	1	0.237
Mie	February 9, 2020	0	0.000
Mie	February 16, 2020	0	0.000
Mie	February 23, 2020	0	0.000
Shiga	February 2, 2020	0	0.000
Shiga	February 9, 2020	0	0.000
Shiga	February 16, 2020	0	0.000
Shiga	February 23, 2020	0	0.000
Kyoto	February 2, 2020	-1	-0.177
Kyoto	February 9, 2020	-1	-0.178
Kyoto	February 16, 2020	0	0.000
Kyoto	February 23, 2020	-1	-0.189
Osaka	February 2, 2020	2	0.109
Osaka	February 9, 2020	0	0.000
Osaka	February 16, 2020	1	0.051
Osaka	February 23, 2020	1	0.055
Hyogo	February 2, 2020	1	0.079
Hyogo	February 9, 2020	-1	-0.085
Hyogo	February 16, 2020	1	0.081
Hyogo	February 23, 2020	0	0.000
Nara	February 2, 2020	-1	-0.294
Nara	February 9, 2020	-1	-0.300
Nara	February 16, 2020	-1	-0.326

Area	Week ending date	Difference*	Percent difference, %
Nara	February 23, 2020	-1	-0.353
Wakayama	February 2, 2020	0	0.000
Wakayama	February 9, 2020	0	0.000
Wakayama	February 16, 2020	0	0.000
Wakayama	February 23, 2020	0	0.000
Tottori	February 2, 2020	1	0.694
Tottori	February 9, 2020	0	0.000
Tottori	February 16, 2020	0	0.000
Tottori	February 23, 2020	0	0.000
Shimane	February 2, 2020	-1	-0.503
Shimane	February 9, 2020	-1	-0.446
Shimane	February 16, 2020	-1	-0.461
Shimane	February 23, 2020	0	0.000
Okayama	February 2, 2020	0	0.000
Okayama	February 9, 2020	0	0.000
Okayama	February 16, 2020	0	0.000
Okayama	February 23, 2020	0	0.000
Hiroshima	February 2, 2020	0	0.000
Hiroshima	February 9, 2020	-1	-0.166
Hiroshima	February 16, 2020	-1	-0.156
Hiroshima	February 23, 2020	0	0.000
Yamaguchi	February 2, 2020	-1	-0.252
Yamaguchi	February 9, 2020	-1	-0.253
Yamaguchi	February 16, 2020	-1	-0.248
Yamaguchi	February 23, 2020	-1	-0.256
Tokushima	February 2, 2020	0	0.000
Tokushima	February 9, 2020	0	0.000
Tokushima	February 16, 2020	0	0.000
Tokushima	February 23, 2020	0	0.000
Kagawa	February 2, 2020	0	0.000
Kagawa	February 9, 2020	0	0.000
Kagawa	February 16, 2020	0	0.000
Kagawa	February 23, 2020	0	0.000
Ehime	February 2, 2020	0	0.000
Ehime	February 9, 2020	0	0.000
Ehime	February 16, 2020	0	0.000
Ehime	February 23, 2020	0	0.000
Kochi	February 2, 2020	-1	-0.426
Kochi	February 9, 2020	-1	-0.433
Kochi	February 16, 2020	-1	-0.431
Kochi	February 23, 2020	-1	-0.498
Fukuoka	February 2, 2020	5	0.441
Fukuoka	February 9, 2020	-1	-0.092
Fukuoka	February 16, 2020	-1	-0.088
Fukuoka	February 23, 2020	1	0.092
Saga	February 2, 2020	0	0.000
Saga	February 9, 2020	0	0.000
Saga	February 16, 2020	0	0.000
Saga	February 23, 2020	0	0.000
Nagasaki	February 2, 2020	1	0.287
Nagasaki	February 9, 2020	0	0.000
Nagasaki	February 16, 2020	0	0.000
Nagasaki	February 23, 2020	0	0.000
Kumamoto	February 2, 2020	0	0.000
Kumamoto	February 9, 2020	1	0.220
Kumamoto	February 16, 2020	0	0.000
Kumamoto	February 23, 2020	0	0.000
Oita	February 2, 2020	0	0.000
Oita	February 9, 2020	0	0.000
Oita	February 16, 2020	0	0.000
Oita	February 23, 2020	0	0.000
Miyazaki	February 2, 2020	0	0.000
Miyazaki	February 9, 2020	0	0.000
Miyazaki	February 16, 2020	1	0.351
Miyazaki	February 23, 2020	0	0.000
Kagoshima	February 2, 2020	0	0.000
Kagoshima	February 9, 2020	0	0.000
Kagoshima	February 16, 2020	1	0.199
Kagoshima	February 23, 2020	0	0.000

Area	Week ending date	Difference*	Percent difference, %
Okinawa	February 2, 2020	-1	-0.403
Okinawa	February 9, 2020	-1	-0.385
Okinawa	February 16, 2020	-1	-0.388
Okinawa	February 23, 2020	-1	-0.389

*Difference is the observed number of all-cause deaths for all ages in February based on up to the May prompt vital statistics with no adjustment for the reporting delay minus those based on up to the April prompt vital statistics with the adjustment for delays in reporting in May. Proportion difference refers to the proportion of the difference to the latter.

Appendix Table 2. Results of sensitivity analyses, excess all-cause death, and percent all-cause excess deaths for all ages, December 30, 2019 through May 31, 2020*

Area	(a) b=3, w=2		(b) b=4, w=2		(c) b=3, w=4		(d) b=4, w=4	
Japan	360–5363	0.06–0.90	358–5307	0.06–0.89	353–5843	0.06–0.98	324–5489	0.05–0.92
Hokkaido	0–95	0.00–0.34	0–109	0.00–0.39	0–115	0.00–0.42	0–134	0.00–0.48
Aomori	0–21	0.00–0.27	0–25	0.00–0.32	0–30	0.00–0.39	0–34	0.00–0.44
Iwate	0–82	0.00–1.08	0–93	0.00–1.23	1–100	0.01–1.32	0–100	0.00–1.32
Miyagi	0–46	0.00–0.43	0–44	0.00–0.41	0–58	0.00–0.54	0–60	0.00–0.56
Akita	0–61	0.00–0.92	0–89	0.00–1.34	0–80	0.00–1.20	0–92	0.00–1.38
Yamagata	0–44	0.00–0.67	0–57	0.00–0.86	0–50	0.00–0.76	0–60	0.00–0.91
Fukushima	0–86	0.00–0.80	0–53	0.00–0.49	0–93	0.00–0.87	0–59	0.00–0.55
Ibaraki	3–107	0.02–0.74	8–107	0.06–0.74	2–102	0.01–0.71	4–97	0.03–0.67
Tochigi	15–159	0.16–1.65	16–167	0.17–1.74	25–177	0.26–1.84	27–180	0.28–1.87
Gunma	25–129	0.25–1.27	35–176	0.34–1.73	39–164	0.38–1.61	46–190	0.45–1.87
Saitama	22–394	0.07–1.29	20–365	0.07–1.20	39–445	0.13–1.46	32–390	0.11–1.28
Chiba	55–277	0.20–1.03	49–241	0.18–0.90	40–289	0.15–1.08	35–247	0.13–0.92
Tokyo	73–459	0.14–0.88	80–438	0.15–0.84	62–466	0.12–0.89	58–424	0.11–0.81
Kanagawa	0–103	0.00–0.28	0–113	0.00–0.31	0–92	0.00–0.25	0–94	0.00–0.26
Niigata	0–0	0.00–0.00	0–0	0.00–0.00	0–5	0.00–0.04	0–0	0.00–0.00
Toyama	39–186	0.69–3.27	33–158	0.58–2.78	36–179	0.63–3.15	28–151	0.49–2.65
Ishikawa	0–50	0.00–0.90	0–60	0.00–1.08	0–57	0.00–1.03	0–67	0.00–1.21
Fukui	0–39	0.00–0.96	0–78	0.00–1.93	0–42	0.00–1.04	0–77	0.00–1.90
Yamanashi	0–52	0.00–1.22	0–52	0.00–1.22	0–65	0.00–1.52	0–52	0.00–1.22
Nagano	0–75	0.00–0.67	0–81	0.00–0.73	0–81	0.00–0.73	0–84	0.00–0.75
Gifu	0–40	0.00–0.40	0–34	0.00–0.34	0–47	0.00–0.48	0–38	0.00–0.38
Shizuoka	17–127	0.09–0.68	15–120	0.08–0.65	11–186	0.06–1.00	11–155	0.06–0.84
Aichi	14–232	0.05–0.76	13–240	0.04–0.78	8–236	0.03–0.77	12–235	0.04–0.77
Mie	0–49	0.00–0.54	0–70	0.00–0.77	0–66	0.00–0.73	0–77	0.00–0.85
Shiga	1–97	0.02–1.73	2–86	0.04–1.53	0–92	0.00–1.64	0–78	0.00–1.39
Kyoto	0–94	0.00–0.80	0–87	0.00–0.74	0–105	0.00–0.89	0–100	0.00–0.85
Osaka	16–280	0.04–0.70	22–295	0.05–0.74	11–284	0.03–0.71	11–271	0.03–0.68
Hyogo	0–108	0.00–0.42	0–106	0.00–0.42	0–135	0.00–0.53	0–118	0.00–0.46
Nara	30–129	0.46–1.99	26–108	0.40–1.67	30–140	0.46–2.16	22–110	0.34–1.70
Wakayama	0–91	0.00–1.64	0–76	0.00–1.37	0–103	0.00–1.86	0–82	0.00–1.48
Tottori	0–34	0.00–1.08	0–39	0.00–1.24	0–45	0.00–1.43	0–45	0.00–1.43
Shimane	5–101	0.12–2.40	0–83	0.00–1.97	2–96	0.05–2.28	0–82	0.00–1.95
Okayama	0–54	0.00–0.57	0–71	0.00–0.75	0–59	0.00–0.62	0–72	0.00–0.76
Hiroshima	0–65	0.00–0.49	0–67	0.00–0.51	0–58	0.00–0.44	0–61	0.00–0.46
Yamaguchi	0–65	0.00–0.80	0–70	0.00–0.86	0–72	0.00–0.88	0–72	0.00–0.88
Tokushima	8–96	0.18–2.21	8–87	0.18–2.01	7–99	0.16–2.28	7–90	0.16–2.07
Kagawa	13–170	0.24–3.16	9–143	0.17–2.66	7–176	0.13–3.28	4–156	0.07–2.90
Ehime	0–90	0.00–1.14	0–80	0.00–1.01	0–110	0.00–1.39	0–94	0.00–1.19
Kochi	2–75	0.05–1.71	2–86	0.05–1.96	1–80	0.02–1.83	0–90	0.00–2.05
Fukuoka	2–159	0.01–0.68	5–142	0.02–0.61	10–148	0.04–0.63	11–124	0.05–0.53
Saga	0–94	0.00–2.13	0–74	0.00–1.68	0–105	0.00–2.38	0–80	0.00–1.81
Nagasaki	0–138	0.00–1.80	0–105	0.00–1.37	0–145	0.00–1.89	0–112	0.00–1.46
Kumamoto	0–118	0.00–1.26	0–128	0.00–1.37	0–131	0.00–1.40	1–138	0.01–1.48
Oita	10–78	0.16–1.24	10–81	0.16–1.29	9–87	0.14–1.39	8–89	0.13–1.42
Miyazaki	10–182	0.16–2.98	5–156	0.08–2.56	13–191	0.21–3.13	7–167	0.11–2.74
Kagoshima	0–77	0.00–0.83	0–107	0.00–1.15	0–86	0.00–0.92	0–102	0.00–1.10
Okinawa	0–55	0.00–1.03	0–60	0.00–1.12	0–71	0.00–1.33	0–59	0.00–1.11

*In the sensitivity analyses, the reference period was changed to confirm the robustness of the results (‘b’ years ago, ‘w’ weeks before and after).

Appendix Table 3. Number of observed and excess all-cause deaths from December 30, 2019 to May 31, 2020, by age groups*

Age group, area	Observed all-cause deaths	Excess all-cause deaths	Percent all-cause excess deaths
(1) under 25 years			
Japan	2915	47–751	1.61–25.76
Hokkaido	103	0–19	0.00–18.45
Aomori	35	0–16	0.00–45.71
Iwate	29	0–14	0.00–48.28
Miyagi	52	1–14	1.92–26.92
Akita	27	2–12	7.41–44.44
Yamagata	26	0–7	0.00–26.92
Fukushima	53	1–19	1.89–35.85
Ibaraki	82	1–25	1.22–30.49
Tochigi	53	0–10	0.00–18.87
Gunma	41	0–10	0.00–24.39
Saitama	148	2–24	1.35–16.22
Chiba	130	1–15	0.77–11.54
Tokyo	262	3–20	1.15–7.63
Kanagawa	168	0–16	0.00–9.52
Niigata	44	0–15	0.00–34.09
Toyama	19	0–6	0.00–31.58
Ishikawa	31	1–14	3.23–45.16
Fukui	29	1–16	3.45–55.17
Yamanashi	14	0–3	0.00–21.43
Nagano	70	0–21	0.00–30.00
Gifu	52	3–22	5.77–42.31
Shizuoka	103	4–33	3.88–32.04
Aichi	198	5–48	2.53–24.24
Mie	53	2–27	3.77–50.94
Shiga	41	2–20	4.88–48.78
Kyoto	47	0–12	0.00–25.53
Osaka	191	0–7	0.00–3.66
Hyogo	127	1–32	0.79–25.20
Nara	34	1–16	2.94–47.06
Wakayama	13	2–6	15.38–46.15
Tottori	11	0–4	0.00–36.36
Shimane	21	2–11	9.52–52.38
Okayama	57	1–23	1.75–40.35
Hiroshima	53	1–12	1.89–22.64
Yamaguchi	31	0–15	0.00–48.39
Tokushima	25	1–13	4.00–52.00
Kagawa	29	1–14	3.45–48.28
Ehime	30	1–12	3.33–40.00
Kochi	22	1–9	4.55–40.91
Fukuoka	114	0–9	0.00–7.89
Saga	17	0–6	0.00–35.29
Nagasaki	36	1–16	2.78–44.44
Kumamoto	51	0–21	0.00–41.18
Oita	22	1–12	4.55–54.55
Miyazaki	28	0–11	0.00–39.29
Kagoshima	52	3–22	5.77–42.31
Okinawa	41	1–22	2.44–53.66
(2) 25–44 years			
Japan	7815	66–1302	0.84–16.66
Hokkaido	344	0–32	0.00–9.30
Aomori	91	0–14	0.00–15.38
Iwate	78	0–18	0.00–23.08
Miyagi	183	4–49	2.19–26.78
Akita	67	1–21	1.49–31.34
Yamagata	88	2–36	2.27–40.91
Fukushima	133	0–22	0.00–16.54
Ibaraki	225	10–54	4.44–24.00
Tochigi	133	0–22	0.00–16.54
Gunma	122	3–21	2.46–17.21
Saitama	450	0–24	0.00–5.33
Chiba	414	0–56	0.00–13.53

Age group, area	Observed all-cause deaths	Excess all-cause deaths	Percent all-cause excess deaths
Tokyo	809	8–67	0.99–8.28
Kanagawa	537	2–61	0.37–11.36
Niigata	130	0–16	0.00–12.31
Toyama	71	0–27	0.00–38.03
Ishikawa	63	6–24	9.52–38.10
Fukui	51	0–18	0.00–35.29
Yamanashi	47	1–19	2.13–40.43
Nagano	108	1–21	0.93–19.44
Gifu	101	1–14	0.99–13.86
Shizuoka	253	4–63	1.58–24.90
Aichi	423	0–30	0.00–7.09
Mie	106	0–28	0.00–26.42
Shiga	82	2–23	2.44–28.05
Kyoto	138	0–25	0.00–18.12
Osaka	542	3–73	0.55–13.47
Hyogo	289	4–39	1.38–13.49
Nara	74	1–18	1.35–24.32
Wakayama	60	0–10	0.00–16.67
Tottori	32	0–9	0.00–28.13
Shimane	45	2–21	4.44–46.67
Okayama	104	0–16	0.00–15.38
Hiroshima	156	1–20	0.64–12.82
Yamaguchi	81	1–28	1.23–34.57
Tokushima	47	0–11	0.00–23.40
Kagawa	65	1–23	1.54–35.38
Ehime	91	0–19	0.00–20.88
Kochi	45	0–17	0.00–37.78
Fukuoka	324	2–46	0.62–14.20
Saga	37	0–8	0.00–21.62
Nagasaki	75	0–15	0.00–20.00
Kumamoto	111	4–40	3.60–36.04
Oita	70	0–15	0.00–21.43
Miyazaki	84	2–31	2.38–36.90
Kagoshima	114	0–19	0.00–16.67
Okinawa	122	0–19	0.00–15.57
(3) 45–64 years			
Japan	44375	207–2958	0.47–6.67
Hokkaido	2118	5–131	0.24–6.19
Aomori	648	10–62	1.54–9.57
Iwate	530	3–44	0.57–8.30
Miyagi	817	4–73	0.49–8.94
Akita	435	0–48	0.00–11.03
Yamagata	432	9–73	2.08–16.90
Fukushima	781	0–37	0.00–4.74
Ibaraki	1120	3–72	0.27–6.43
Tochigi	794	13–77	1.64–9.70
Gunma	645	0–29	0.00–4.50
Saitama	2716	12–212	0.44–7.81
Chiba	2226	0–56	0.00–2.52
Tokyo	4579	23–183	0.50–4.00
Kanagawa	3054	11–162	0.36–5.30
Niigata	777	0–26	0.00–3.35
Toyama	332	1–18	0.30–5.42
Ishikawa	335	1–38	0.30–11.34
Fukui	240	7–43	2.92–17.92
Yamanashi	297	0–25	0.00–8.42
Nagano	639	0–47	0.00–7.36
Gifu	623	0–40	0.00–6.42
Shizuoka	1268	4–101	0.32–7.97
Aichi	2337	11–104	0.47–4.45
Mie	592	5–57	0.84–9.63
Shiga	341	0–19	0.00–5.57
Kyoto	736	0–19	0.00–2.58
Osaka	3178	32–156	1.01–4.91
Hyogo	1767	9–68	0.51–3.85
Nara	449	0–38	0.00–8.46
Wakayama	355	0–40	0.00–11.27

Age group, area	Observed all-cause deaths	Excess all-cause deaths	Percent all-cause excess deaths
Tottori	212	5–42	2.36–19.81
Shimane	219	0–21	0.00–9.59
Okayama	551	0–28	0.00–5.08
Hiroshima	926	2–71	0.22–7.67
Yamaguchi	532	8–75	1.50–14.10
Tokushima	270	0–32	0.00–11.85
Kagawa	328	0–39	0.00–11.89
Ehime	528	0–39	0.00–7.39
Kochi	281	13–51	4.63–18.15
Fukuoka	1740	9–89	0.52–5.11
Saga	316	0–43	0.00–13.61
Nagasaki	554	1–75	0.18–13.54
Kumamoto	635	0–69	0.00–10.87
Oita	339	0–6	0.00–1.77
Miyazaki	457	6–75	1.31–16.41
Kagoshima	686	0–79	0.00–11.52
Okinawa	640	0–26	0.00–4.06
(4) 65–74 years			
Japan	85102	143–2959	0.17–3.48
Hokkaido	4192	8–131	0.19–3.13
Aomori	1169	5–45	0.43–3.85
Iwate	1026	3–64	0.29–6.24
Miyagi	1492	0–59	0.00–3.95
Akita	877	4–89	0.46–10.15
Yamagata	776	0–26	0.00–3.35
Fukushima	1528	6–108	0.39–7.07
Ibaraki	2181	0–56	0.00–2.57
Tochigi	1480	1–56	0.07–3.78
Gunma	1491	0–56	0.00–3.76
Saitama	4951	0–113	0.00–2.28
Chiba	4143	2–77	0.05–1.86
Tokyo	7451	22–139	0.30–1.87
Kanagawa	5236	0–50	0.00–0.95
Niigata	1642	0–56	0.00–3.41
Toyama	755	2–50	0.26–6.62
Ishikawa	773	20–85	2.59–11.00
Fukui	500	1–56	0.20–11.20
Yamanashi	508	0–43	0.00–8.46
Nagano	1323	4–90	0.30–6.80
Gifu	1272	2–35	0.16–2.75
Shizuoka	2619	0–57	0.00–2.18
Aichi	4534	5–107	0.11–2.36
Mie	1160	0–24	0.00–2.07
Shiga	750	0–32	0.00–4.27
Kyoto	1613	0–67	0.00–4.15
Osaka	6312	5–105	0.08–1.66
Hyogo	3554	11–97	0.31–2.73
Nara	812	9–34	1.11–4.19
Wakayama	757	1–70	0.13–9.25
Tottori	440	0–44	0.00–10.00
Shimane	507	0–54	0.00–10.65
Okayama	1320	14–108	1.06–8.18
Hiroshima	1853	6–66	0.32–3.56
Yamaguchi	1152	2–65	0.17–5.64
Tokushima	580	0–53	0.00–9.14
Kagawa	761	2–79	0.26–10.38
Ehime	1101	0–28	0.00–2.54
Kochi	586	0–19	0.00–3.24
Fukuoka	3493	0–75	0.00–2.15
Saga	575	0–15	0.00–2.61
Nagasaki	1022	0–15	0.00–1.47
Kumamoto	1177	4–64	0.34–5.44
Oita	805	2–51	0.25–6.34
Miyazaki	781	0–36	0.00–4.61
Kagoshima	1245	0–63	0.00–5.06
Okinawa	827	2–47	0.24–5.68

Age group, area	Observed all-cause deaths	Excess all-cause deaths	Percent all-cause excess deaths
(5) 75–84 years			
Japan	166547	110–3100	0.07–1.86
Hokkaido	7635	0–86	0.00–1.13
Aomori	2157	3–69	0.14–3.20
Iwate	1991	0–63	0.00–3.16
Miyagi	2854	4–76	0.14–2.66
Akita	1702	5–107	0.29–6.29
Yamagata	1584	0–56	0.00–3.54
Fukushima	2654	3–41	0.11–1.54
Ibaraki	4059	0–38	0.00–0.94
Tochigi	2654	10–121	0.38–4.56
Gunma	2776	15–75	0.54–2.70
Saitama	9697	0–49	0.00–0.51
Chiba	8403	12–192	0.14–2.28
Tokyo	15123	0–86	0.00–0.57
Kanagawa	10953	0–31	0.00–0.28
Niigata	3172	0–18	0.00–0.57
Toyama	1524	2–90	0.13–5.91
Ishikawa	1437	5–57	0.35–3.97
Fukui	985	0–9	0.00–0.91
Yamanashi	1147	3–78	0.26–6.80
Nagano	2665	2–61	0.08–2.29
Gifu	2794	0–35	0.00–1.25
Shizuoka	5135	0–70	0.00–1.36
Aichi	9345	0–65	0.00–0.70
Mie	2513	0–37	0.00–1.47
Shiga	1495	10–60	0.67–4.01
Kyoto	3435	0–83	0.00–2.42
Osaka	12792	0–152	0.00–1.19
Hyogo	7320	0–88	0.00–1.20
Nara	1838	8–54	0.44–2.94
Wakayama	1509	0–31	0.00–2.05
Tottori	724	0–33	0.00–4.56
Shimane	983	5–66	0.51–6.71
Okayama	2432	0–62	0.00–2.55
Hiroshima	3535	0–76	0.00–2.15
Yamaguchi	2148	0–64	0.00–2.98
Tokushima	1075	7–48	0.65–4.47
Kagawa	1330	0–51	0.00–3.83
Ehime	2021	0–72	0.00–3.56
Kochi	1068	2–69	0.19–6.46
Fukuoka	6191	0–27	0.00–0.44
Saga	1085	2–54	0.18–4.98
Nagasaki	1959	6–95	0.31–4.85
Kumamoto	2119	0–44	0.00–2.08
Oita	1561	0–75	0.00–4.80
Miyazaki	1470	5–68	0.34–4.63
Kagoshima	2167	1–58	0.05–2.68
Okinawa	1331	0–60	0.00–4.51
(6) 85 years or older			
Japan	291637	73–2466	0.03–0.85
Hokkaido	13311	0–32	0.00–0.24
Aomori	3671	0–23	0.00–0.63
Iwate	3987	0–37	0.00–0.93
Miyagi	5384	0–26	0.00–0.48
Akita	3608	0–3	0.00–0.08
Yamagata	3737	0–39	0.00–1.04
Fukushima	5599	0–23	0.00–0.41
Ibaraki	6834	0–43	0.00–0.63
Tochigi	4565	0–39	0.00–0.85
Gunma	5135	5–108	0.10–2.10
Saitama	12512	0–129	0.00–1.03
Chiba	11583	21–185	0.18–1.60
Tokyo	24170	26–200	0.11–0.83
Kanagawa	16272	0–46	0.00–0.28
Niigata	7001	0–31	0.00–0.44
Toyama	3046	4–63	0.13–2.07

Age group, area	Observed all-cause deaths	Excess all-cause deaths	Percent all-cause excess deaths
Ishikawa	2938	0–24	0.00–0.82
Fukui	2274	7–34	0.31–1.50
Yamanashi	2316	6–40	0.26–1.73
Nagano	6378	0–11	0.00–0.17
Gifu	5106	0–37	0.00–0.72
Shizuoka	9232	0–42	0.00–0.45
Aichi	13794	0–106	0.00–0.77
Mie	4666	0–42	0.00–0.90
Shiga	2929	2–49	0.07–1.67
Kyoto	5900	0–51	0.00–0.86
Osaka	17046	0–104	0.00–0.61
Hyogo	12481	0–71	0.00–0.57
Nara	3300	0–69	0.00–2.09
Wakayama	2888	0–49	0.00–1.70
Tottori	1769	0–46	0.00–2.60
Shimane	2488	0–58	0.00–2.33
Okayama	5089	0–39	0.00–0.77
Hiroshima	6781	0–7	0.00–0.10
Yamaguchi	4261	0–7	0.00–0.16
Tokushima	2362	0–36	0.00–1.52
Kagawa	2903	2–78	0.07–2.69
Ehime	4201	0–16	0.00–0.38
Kochi	2417	0–40	0.00–1.65
Fukuoka	11542	0–141	0.00–1.22
Saga	2415	0–39	0.00–1.61
Nagasaki	4076	0–28	0.00–0.69
Kumamoto	5311	0–10	0.00–0.19
Oita	3521	0–29	0.00–0.82
Miyazaki	3339	0–78	0.00–2.34
Kagoshima	5102	0–9	0.00–0.18
Okinawa	2397	0–49	0.00–2.04

*The national-level cumulative number of excess all-cause deaths was calculated by summing those of 47 prefectures. Due to the fact that the adjusted number of deaths was rounded up to the integer, the sum of the values by age groups does not necessarily equal that of all ages.

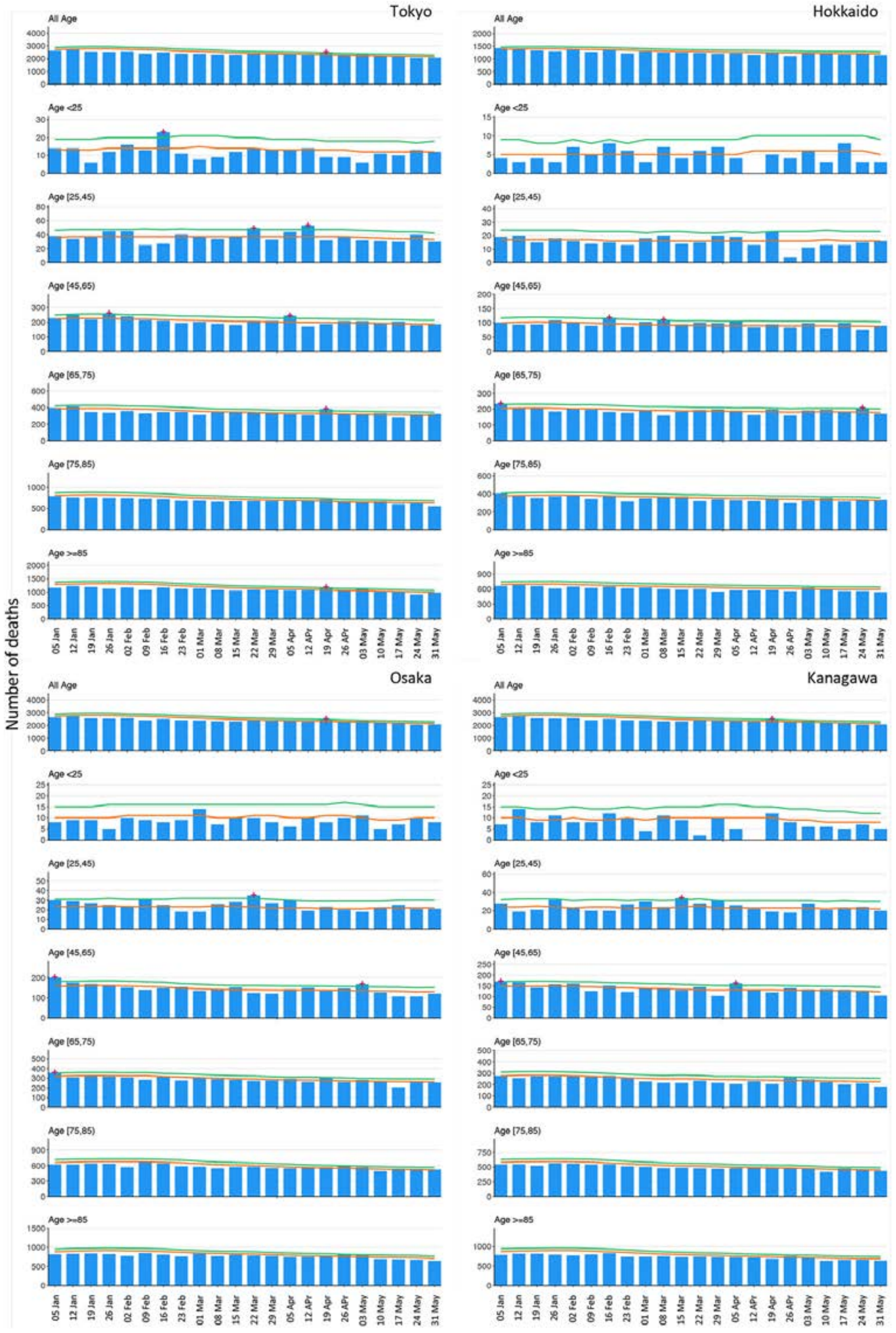

Appendix Figure 1. Observed and expected number of all-cause deaths in four prefectures with large number of reported COVID19 deaths for all ages and by age group, from January 2020 (December 30, 2019 to January 5, 2020) to May 2020 (May 25 to 31, 2020). Blue: observed; Green: upper bound;

Orange: point estimate; cross symbols indicate weeks with the observed exceeding the 95% upper bound. To the national-level data, the observed and expected number of all-cause deaths for each prefecture were summed for each week, which were then used to calculate the weekly national-level excess deaths.

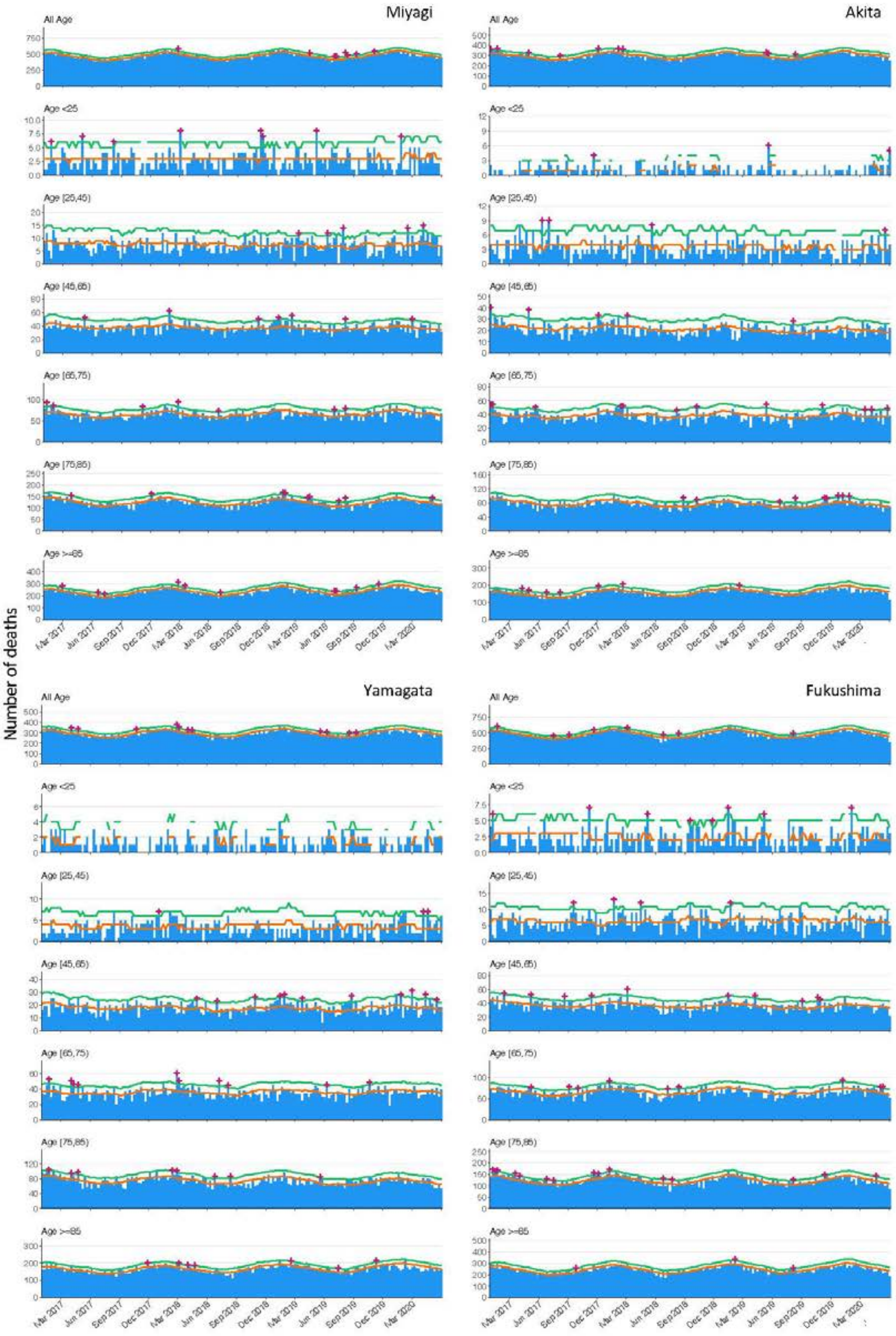
Miyagi
Akita
Yamagata
Fukushima
Number of deaths
All Age
Age <25
Age [25,45)
Age [45,65)
Age [65,75)
Age [75,85)
Age >=85
Mar 2017
Jun 2017
Sep 2017
Dec 2017
Mar 2018
Jun 2018
Sep 2018
Dec 2018
Mar 2019
Jun 2019
Sep 2019
Dec 2019
Mar 2020

3

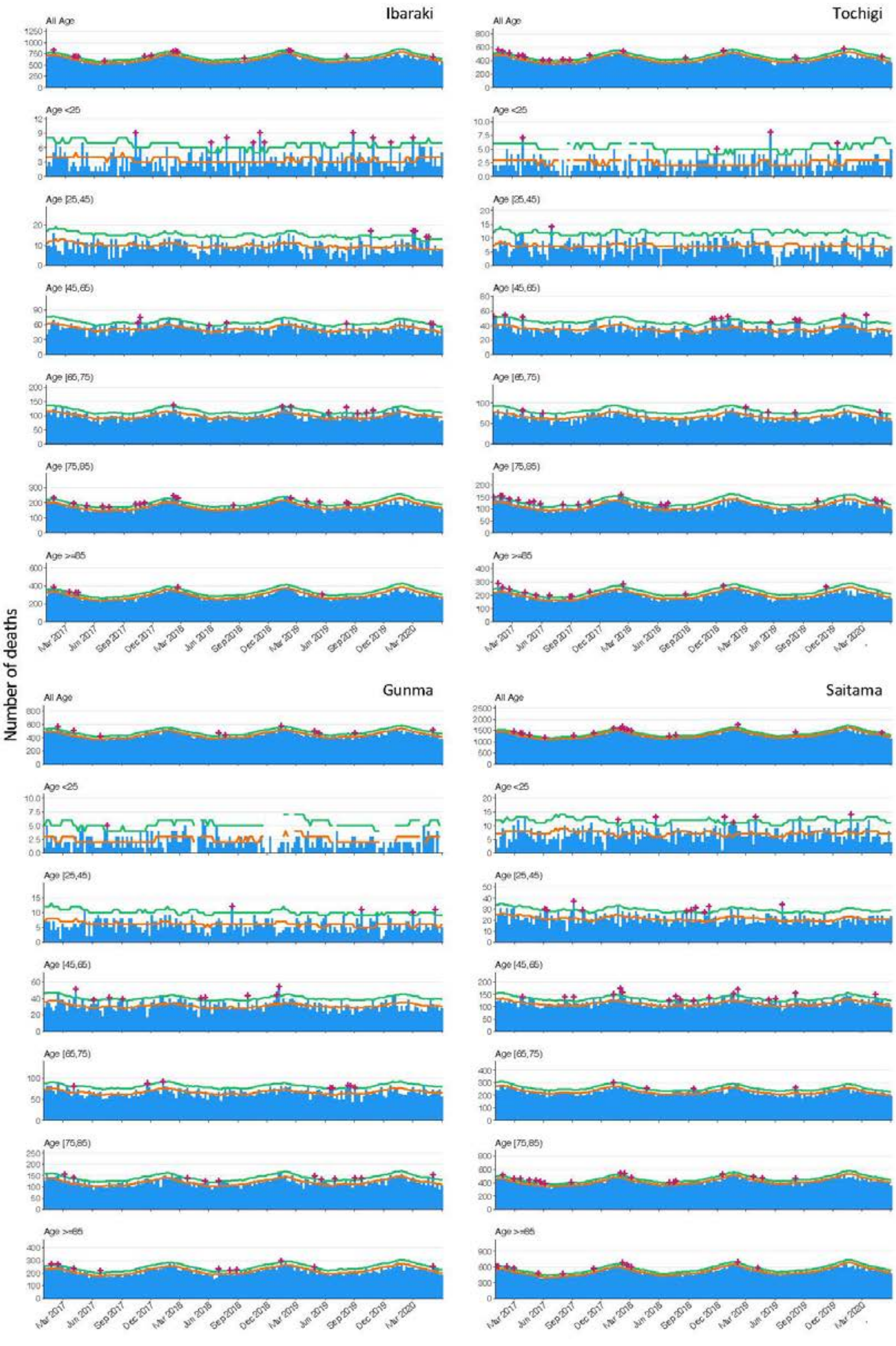
Ibaraki
Tochigi
Gunma
Saitama
Number of deaths
All Age
Age <25
Age [25,45)
Age [45,65)
Age [65,75)
Age [75,85)
Age >=85
Mar 2017
Jun 2017
Sep 2017
Dec 2017
Mar 2018
Jun 2018
Sep 2018
Dec 2018
Mar 2019
Jun 2019
Sep 2019
Dec 2019
Mar 2020

4

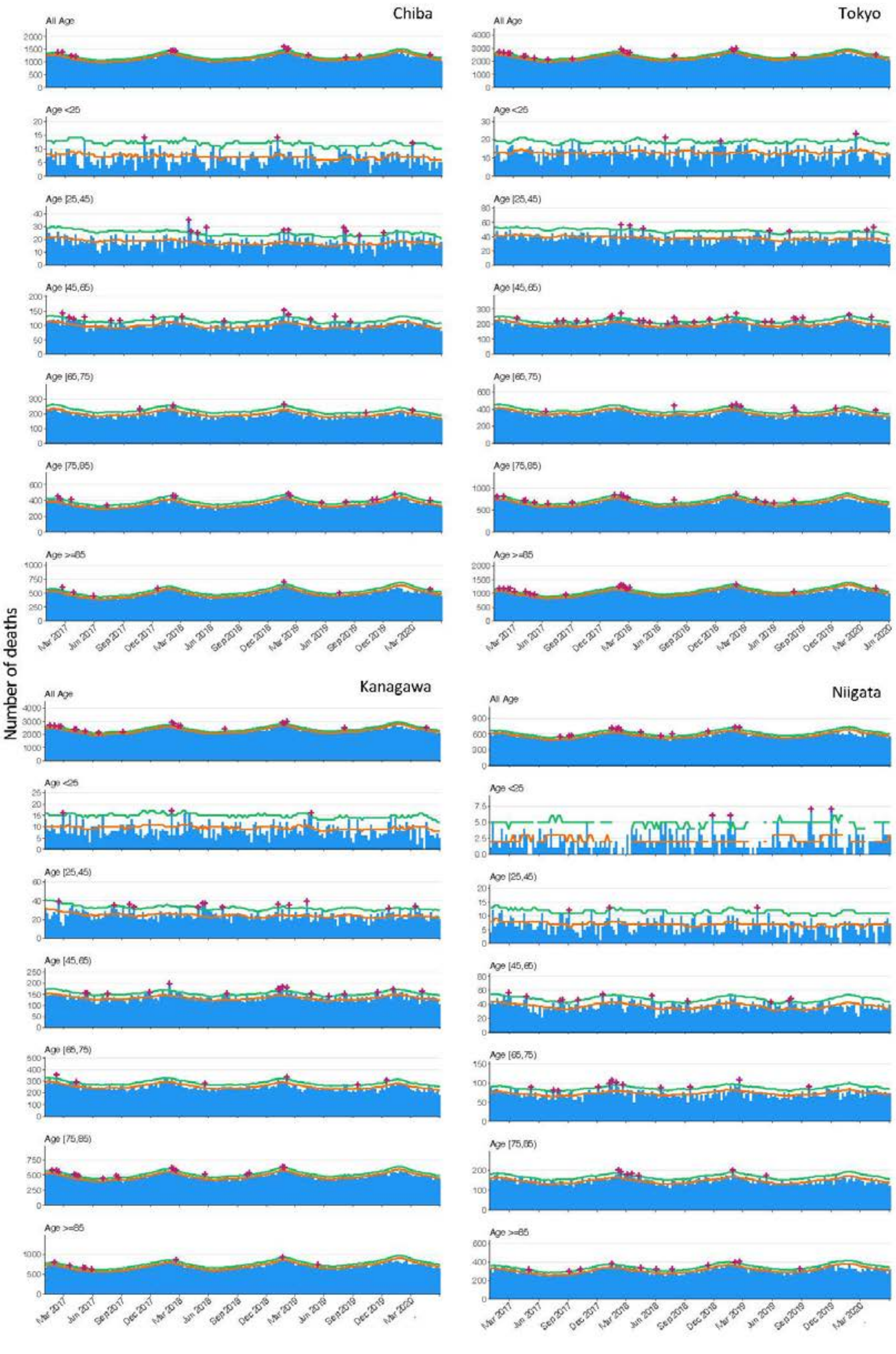
Chiba
Tokyo
Kanagawa
Niigata
Number of deaths
All Age
Age <25
Age [25,45)
Age [45,65)
Age [65,75)
Age [75,85)
Age >=85

5

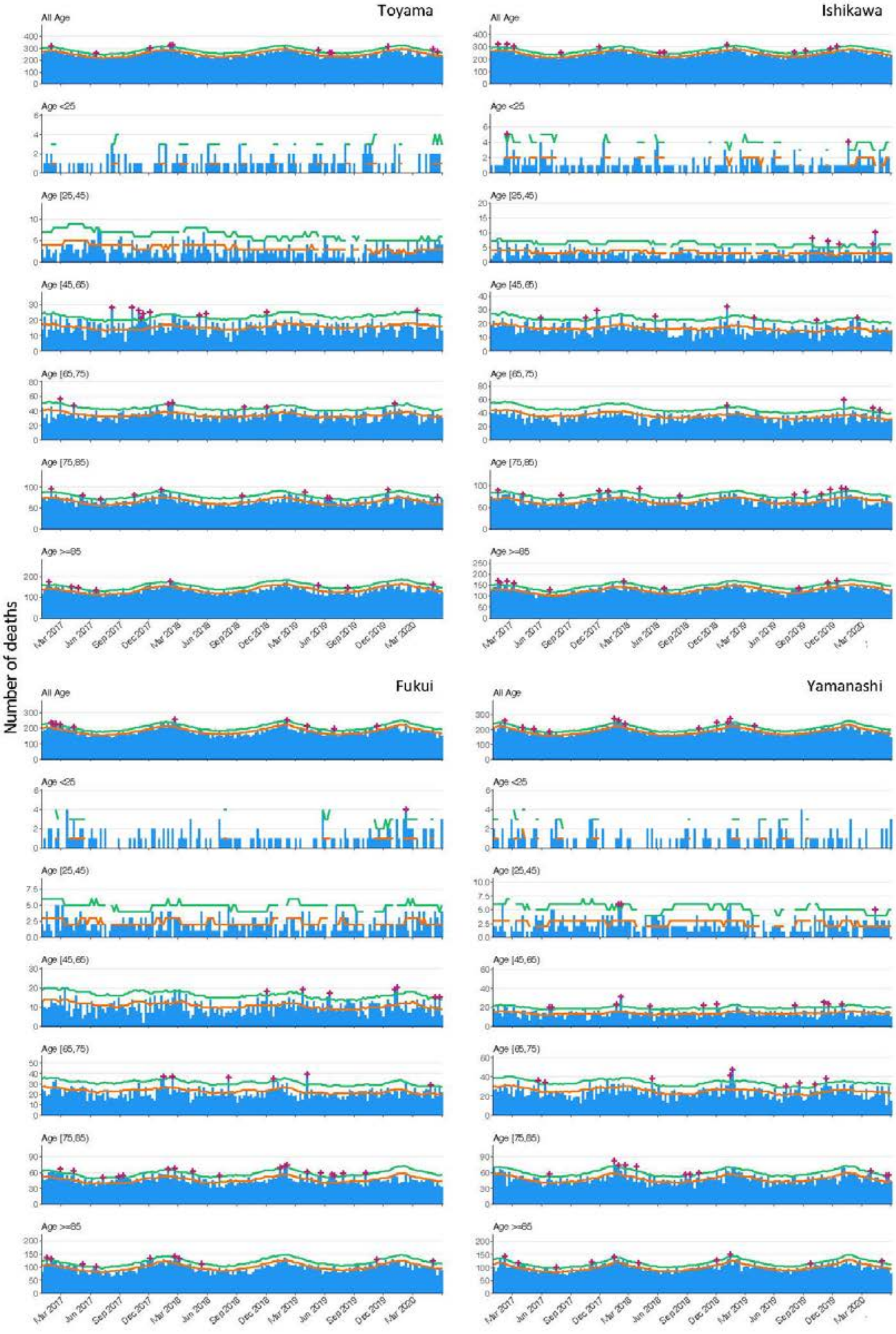
Toyama
Ishikawa
Fukui
Yamanashi
All Age
Age <25
Age [25,45)
Age [45,65)
Age [65,75)
Age [75,85)
Age >=85
Number of deaths
Mar 2017
Jun 2017
Sep 2017
Dec 2017
Mar 2018
Jun 2018
Sep 2018
Dec 2018
Mar 2019
Jun 2019
Sep 2019
Dec 2019
Mar 2020

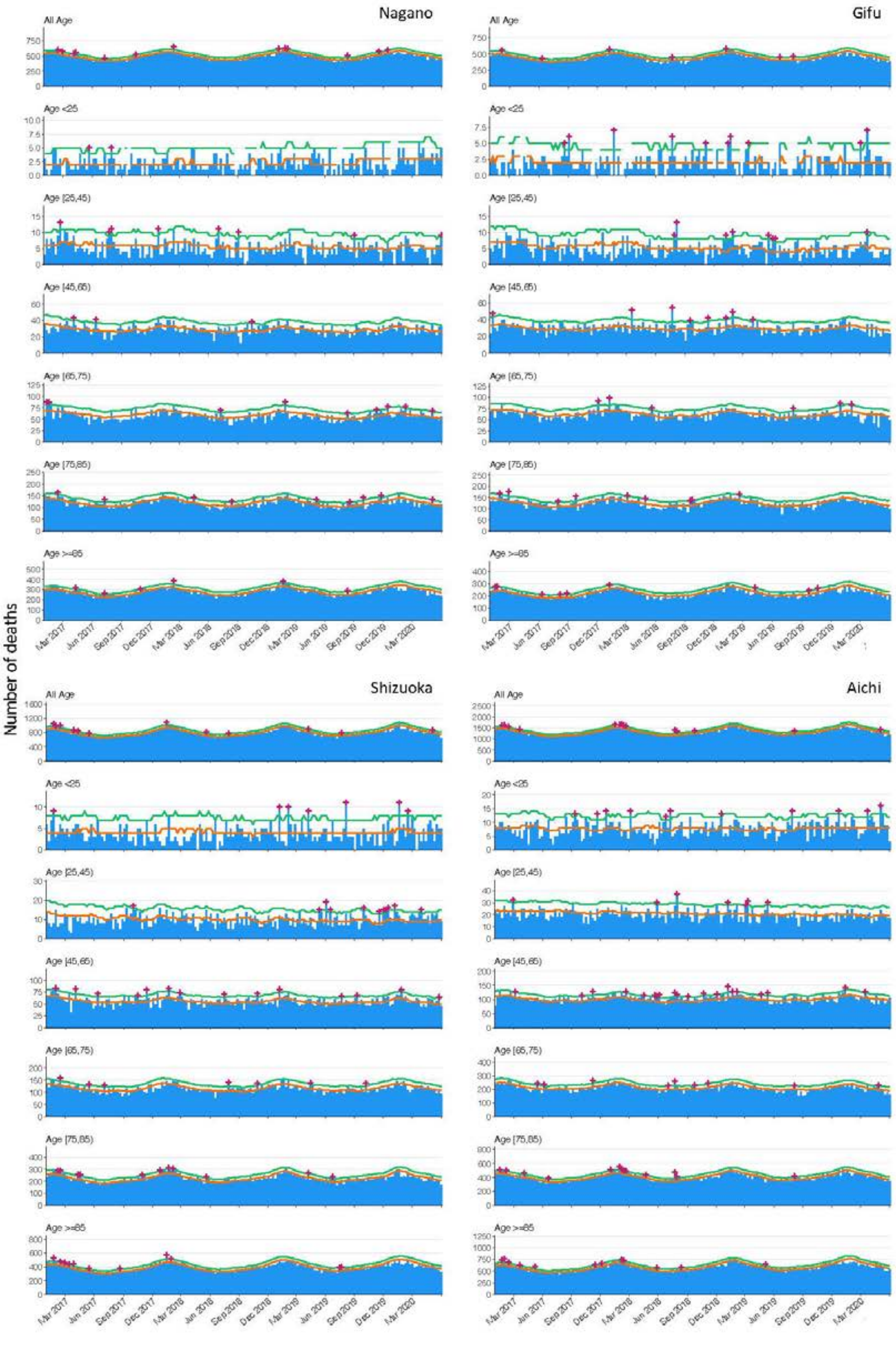
Nagano
Gifu
Shizuoka
Aichi
Number of deaths
All Age
Age <25
Age [25,45)
Age [45,65)
Age [65,75)
Age [75,85)
Age >=85
Mar 2017
Jun 2017
Sep 2017
Dec 2017
Mar 2018
Jun 2018
Sep 2018
Dec 2018
Mar 2019
Jun 2019
Sep 2019
Dec 2019
Mar 2020

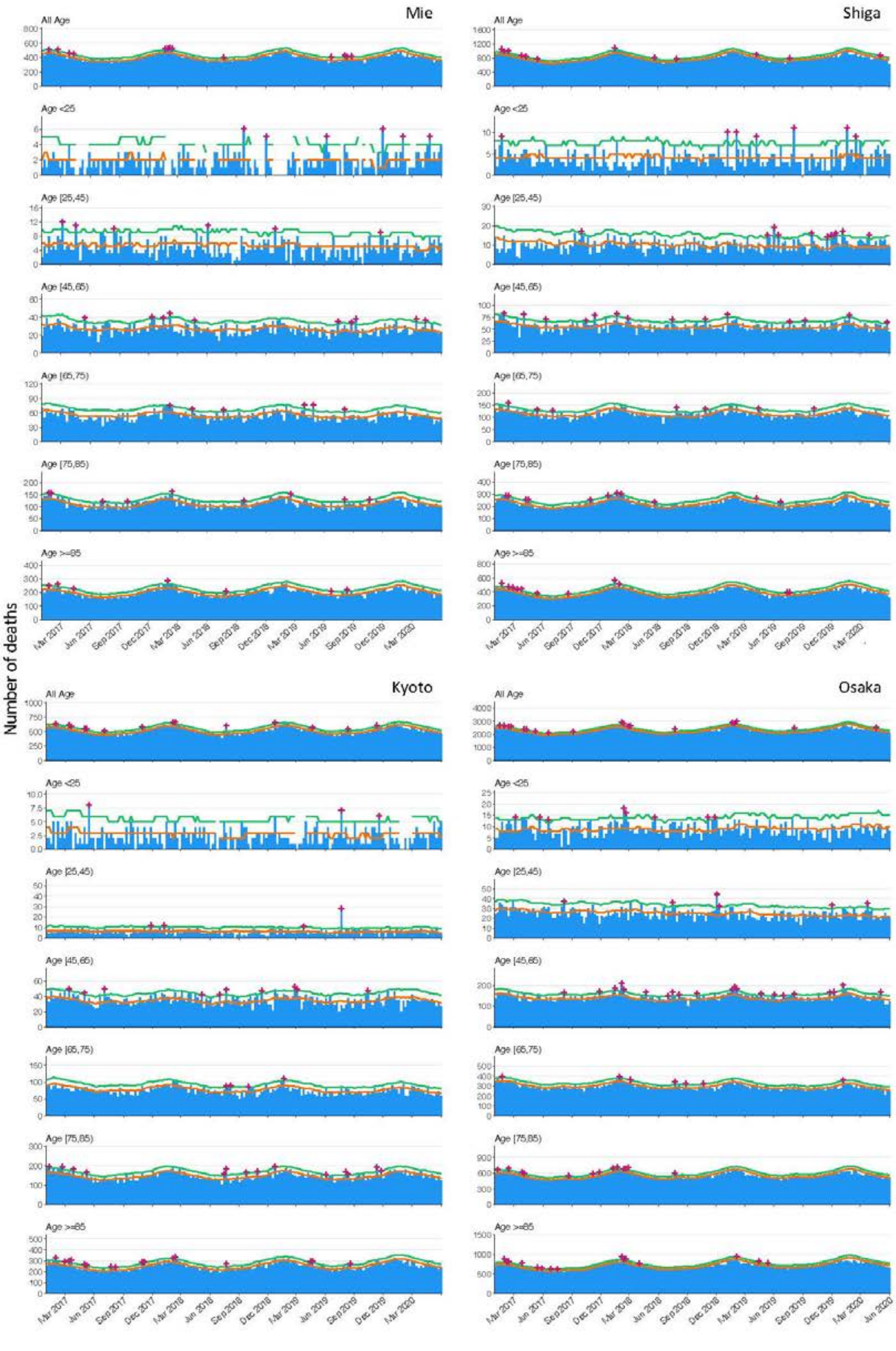
Mie
Shiga
Kyoto
Osaka
Number of deaths
All Age
Age <25
Age [25,45)
Age [45,65)
Age [65,75)
Age [75,85)
Age >=85

8

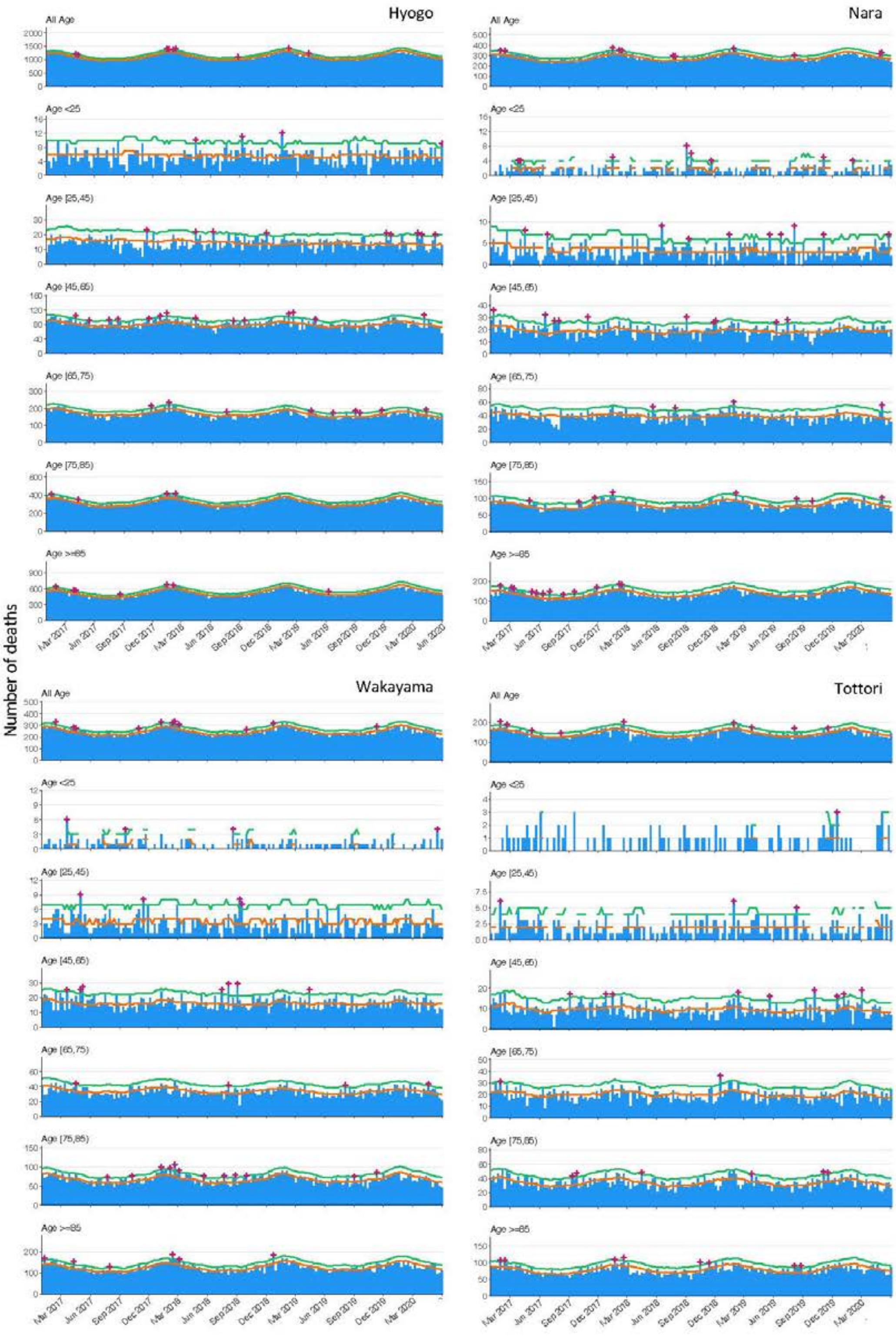
Hyogo
Nara
Wakayama
Tottori
Number of deaths
All Age
Age <25
Age [25,45)
Age [45,65)
Age [65,75)
Age [75,85)
Age >=85

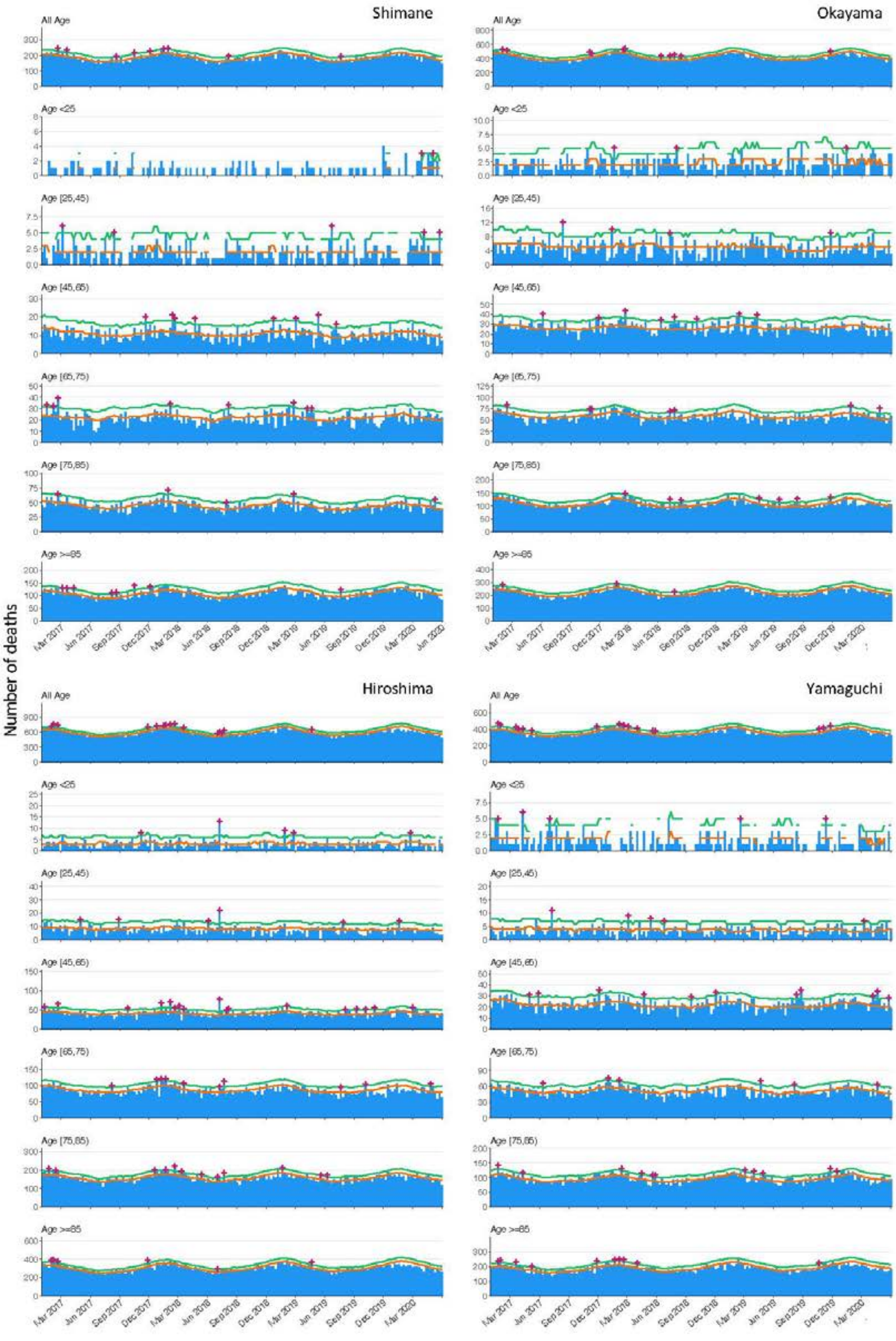
Shimane
Okayama
Hiroshima
Yamaguchi
Number of deaths
All Age
Age <25
Age [25,45)
Age [45,65)
Age [65,75)
Age [75,85)
Age >=85

10

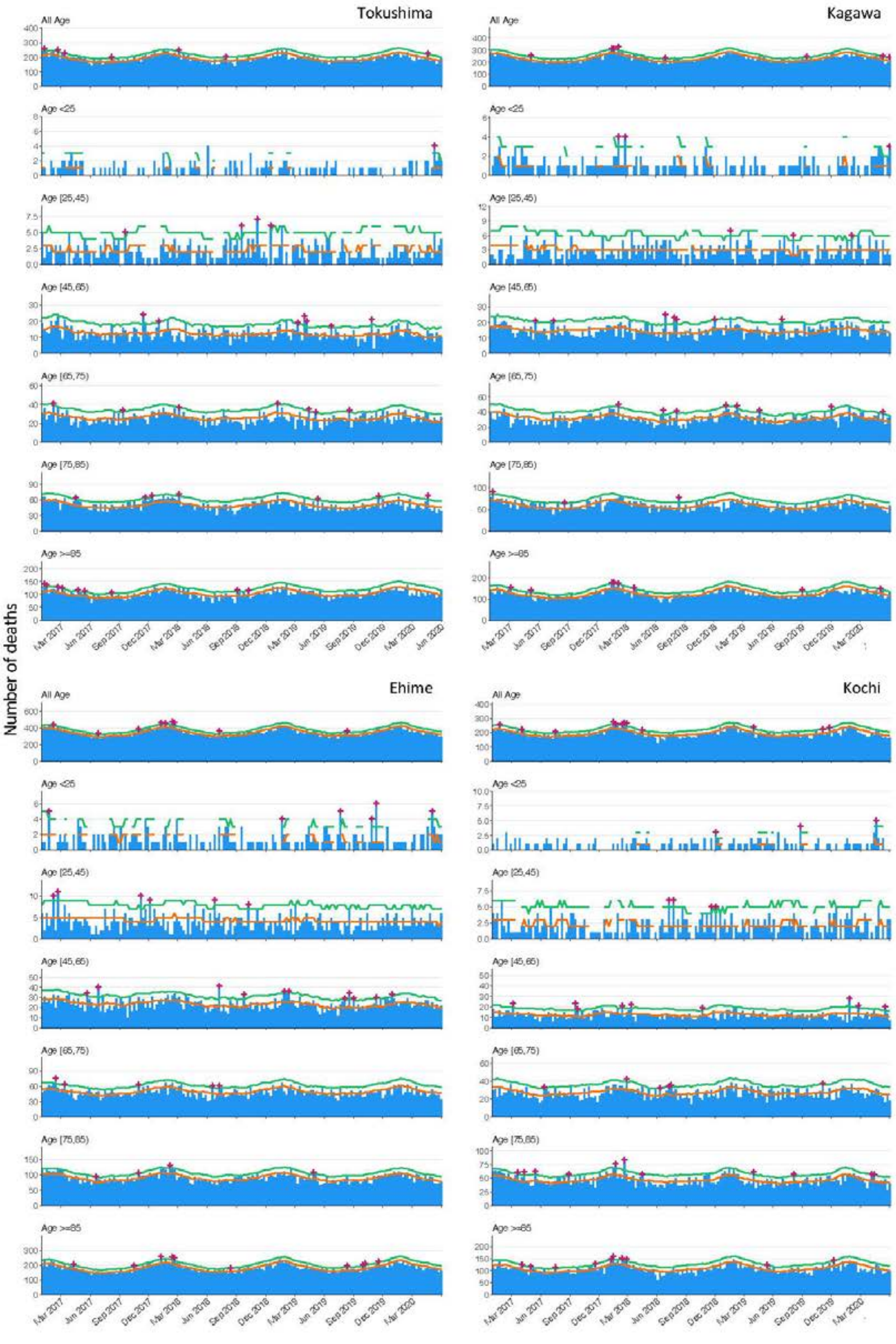
Tokushima
Kagawa
Ehime
Kochi
Number of deaths
All Age
Age <25
Age [25,45)
Age [45,65)
Age [65,75)
Age [75,85)
Age >=85
Mar 2017
Jun 2017
Sep 2017
Dec 2017
Mar 2018
Jun 2018
Sep 2018
Dec 2018
Mar 2019
Jun 2019
Sep 2019
Dec 2019
Mar 2020
Jun 2020

11

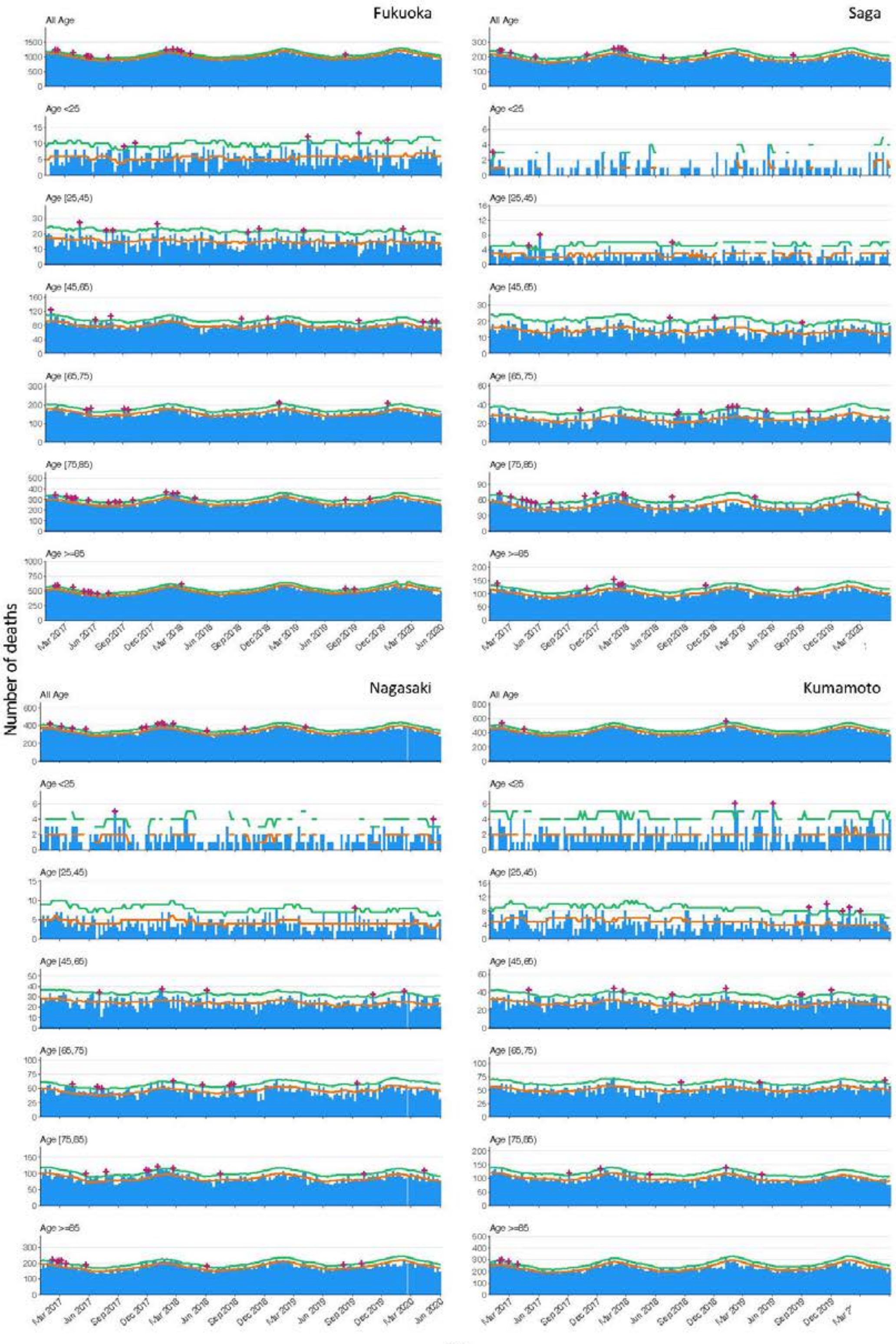
Fukuoka
Saga
Nagasaki
Kumamoto
Number of deaths
All Age
Age <25
Age [25,45)
Age [45,65)
Age [65,75)
Age [75,85)
Age >=85

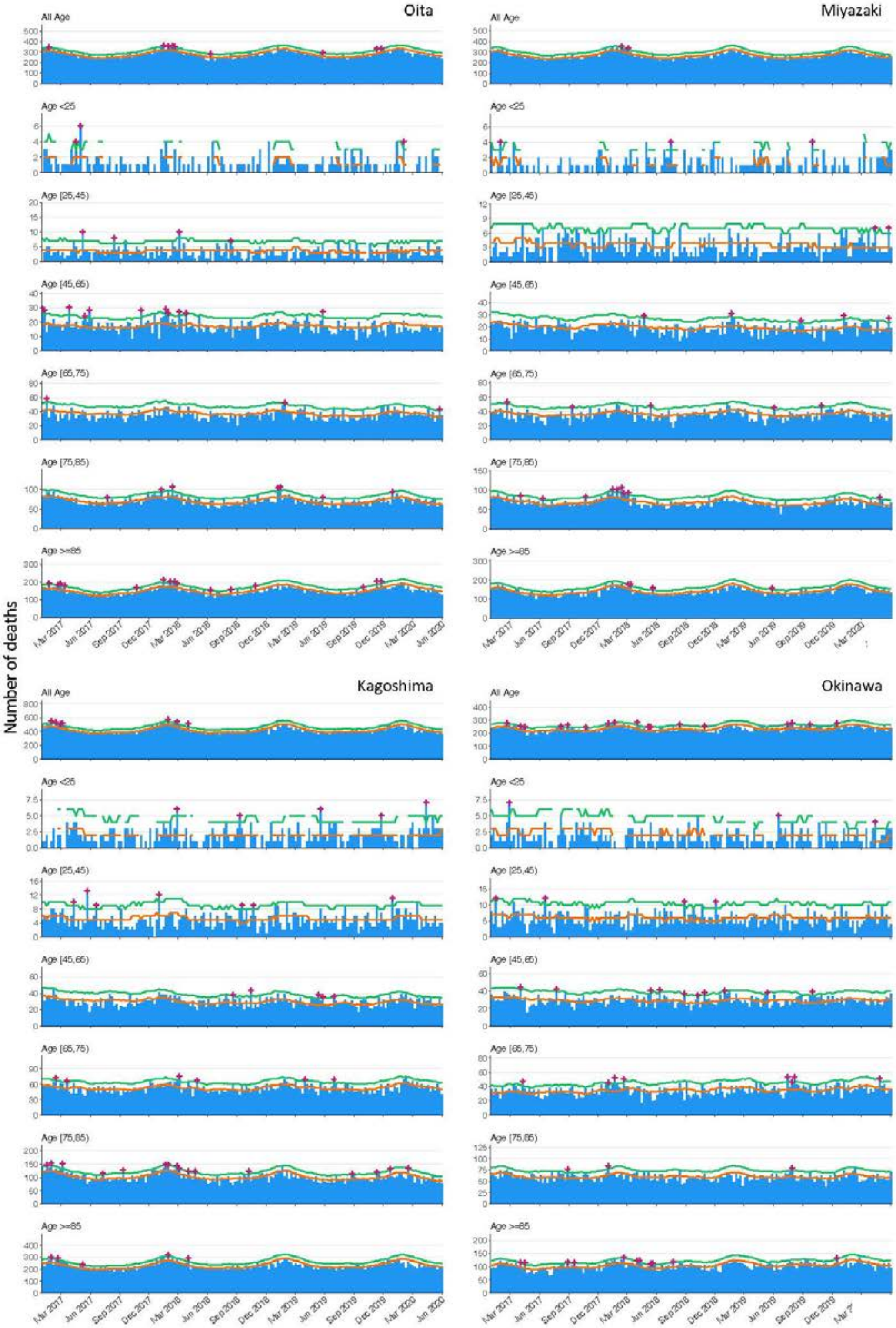
Oita
Miyazaki
Kagoshima
Okinawa
Number of deaths
All Age
Age <25
Age [25,45)
Age [45,65)
Age [65,75)
Age [75,85)
Age >=85

13

Appendix Figure 2. Observed and expected number of all-cause deaths in the national level and 47 prefectures for all ages and by age group, from January 2017 (December 26, 2016 to January 1, 2017) to May 2020 (May 25 to 31, 2020). Blue: observed; Green: upper bound; Orange: point estimate; cross symbols indicate weeks with the observed exceeding the 95% upper bound. To obtain the national-level data, the observed and expected number of all-cause deaths for each prefecture were summed for each week, which were then used to calculate the weekly national-level excess deaths.
